# Migration, adaptation, innovation: The spread of Neolithic harvesting technologies in the Mediterranean

**DOI:** 10.1371/journal.pone.0232455

**Published:** 2020-04-30

**Authors:** Niccolò Mazzucco, Juan José Ibáñez, Giacomo Capuzzo, Bernard Gassin, Mario Mineo, Juan Francisco Gibaja

**Affiliations:** 1 Archaeology of Social Dynamics (ASD), Spanish National Research Council (CSIC), Institución Milá y Fontanals (IMF), Barcelona, Spain; 2 Laboratory of Anthropology and Human Genetics, Faculty of Science, Université Libre de Bruxelles, Brussels, Belgium; 3 UMR 5608, TRACES, CNRS / Université de Toulouse II-Le Mirail, Toulouse, France; 4 Museo delle Civiltà / Museo Nazionale Preistorico Etnografico ‘L. Pigorini’, Roma, Italia; University at Buffalo - The State University of New York, UNITED STATES

## Abstract

This article explores the changes that occurred in harvesting technology during the dispersal of the Neolithic in the Mediterranean basin. It does so through technological and use-wear analysis of flaked stone tools from archaeological sites dated between ca. 7000 and 5000 cal BCE, from the Aegean Sea to the westernmost coasts of Portugal. The main goal is to analyse the transformations that occurred in the harvesting toolkit. Our study reveals dynamics of continuity and change in sickles at a Mediterranean scale, resulting from adaptations of the migrant groups to the newly occupied territories and from processes of technological innovation. Adaptations in the production system of the inserts and in their use-pattern occurred in relation to lithic raw material availability and knappers’ skills, but also in relation to the scale of production and farming techniques. A major shift took place in the north-western Mediterranean arc with the diffusion of parallel-hafted inserts, probably as a result of heterogeneous phenomena including the diffusion of new groups, technical transfers, establishment of new interaction networks and new systems of lithic production.

## 1. Introduction

Human migrations are complex phenomena that involve contrasting patterns of resilience and innovation, continuity and transformation, adaptation and rigidity, acculturation and assimilation. Individuals and social groups that move from one specific location to a new one face changing conditions that might constitute opportunities as well as constraints for adaptation, including variations in the environmental setting, in the demographic structure of the society, in its level of technological development, and in its forms of political organisation [1,2,3]. Understanding how migrant communities react to such challenges is currently an important topic of research for anthropologists, sociologists and psychologists focused on the contemporary world, but it equally represents a crucial question for historians and archaeologists aiming to reconstruct past migration processes and their consequences [4–10].

The spread of the Neolithic has been one of the most debated examples of past migration [11,12,13]. The discussion has long been dominated by two contrasting visions, opposing the demic diffusion of colonist peasants to the adoption of farming by indigenous hunter-gatherer populations [14–19], even if intermediate models have also been suggested [20,21,22]. The variability of the material record recovered from sites in the Mediterranean Basin and in Eastern and Central Europe has often been interpreted in terms of ‘Mesolithic inheritances’ as opposed to a ‘Neolithic package’. For example, discontinuities in the lithic and ceramic record were often claimed to represent proof of a recomposition of the package, integrating hunter-gatherer technical traditions as a result of interactions between groups and/or of the adoption by local hunter-gatherers of the agro-pastoral way of life [23–28 among many others]. While several efforts have been made to explore farmer-forager interactions, the occurrence of internal, spontaneous transformations within Neolithic farming societies are much less acknowledged. Nevertheless, during their diffusion across the Mediterranean and Europe, Neolithic societies experienced important changes in their demographic and social composition, in the environmental and climate conditions they faced, and in resources and raw-material availability. Some authors have recently proposed that important shifts took place in the farming package during the northward dispersal from the Aegean area towards the interior of the Balkans and continental Europe, due to climate-related adaptations in the modes of exploitation of domesticated plants and animals. It is now widely accepted that Neolithic technologies did not diffuse as a unique package, but followed distinct dynamics and evolutionary paths [29–32].

At the time Neolithic populations started to spread in the Mediterranean they had already faced important changes in all aspects of society. Whereas the first wave of cultivators spread to Cyprus during the 10^th^-9^th^ millennia BCE [33], the Neolithic expanded further across the Mediterranean Basin between 4,000 and 2,000 years after the emergence of the first cereal cultivation in the Pre-Pottery Neolithic A [22, 34–38]. During this long period, harvesting technologies passed through several transformations, concerning both the methods of production of the stone inserts used to form the cutting edge of the harvesting tools [39–42] and the shape and mode of usage of the harvesting tools themselves [43–48]. At the beginning of the 7th millennium BCE, several types of harvesting tools coexisted between Anatolia, the Fertile Crescent and Cyprus, including curved sickles with both straight and serrated cutting edges and reaping knives with a single blade. Inserts were manufactured by a variety of technological systems, including bidirectional blade production, pressure technique, and less skilled flaking systems (i.e. direct percussion with hard hammer, flake production, etc.). Therefore, a variety of technological options for both insert production and maintenance were available to the first seafaring farmers. Not only a broad array of flaking techniques but also a diversity of lithic raw materials were available to produce harvesting inserts. Chert was generally preferred over obsidian for sickle blade production, even in regions where obsidian is dominant, for example Central Anatolia, at least in certain periods [49,50]. This might be related to the greater fragility of obsidian, which quickly causes the loss of the cutting edge and therefore reduction in the sickle’s efficiency, as also experimentally tested [51].

This paper explores the changes that occurred in harvesting technology during the dispersal of the Neolithic in the Mediterranean basin. A comparative technological and use-wear analysis of the ‘glossy tools’ has been carried out at a Mediterranean scale. This approach has been previously used to define the type of harvesting tools and their evolution in specific areas [52–57]. The present article clarifies regional differences in the Neolithic harvesting toolkit at a Mediterranean scale and refines the chronologies of its diffusion. The main objective is to discuss whether observed transformations in the agricultural toolkit were related to adaptations of the migrant groups to the newly occupied territories and/or to the process of technological innovation.

The study of harvesting technologies is particularly relevant to this goal. Harvesting is a key operation within agricultural production. It is a labour-intensive and time-critical task; performing it at the right time maximises yield and minimises grain loss and deterioration. However, the use of a determinate harvesting technology depends on a diversity of technical, environmental, sociocultural and economic aspects. The size of the agricultural fields, the cultivated species, the type of soils, the agents involved in the harvesting tasks, the availability of raw materials for tool manufacture and maintenance, as well as political, religious and symbolic aspects; all of these elements influence the adoption of one technique or another [58–62]. Variations in the harvesting toolkit can therefore provide relevant information on the Neolithic farming system and its adaptation throughout the process of expansion. Cultural, economic and technical factors might have affected the way in which harvesting tools were produced and used by the first farmers.

## 2. Materials and methods

**Fig 1:**
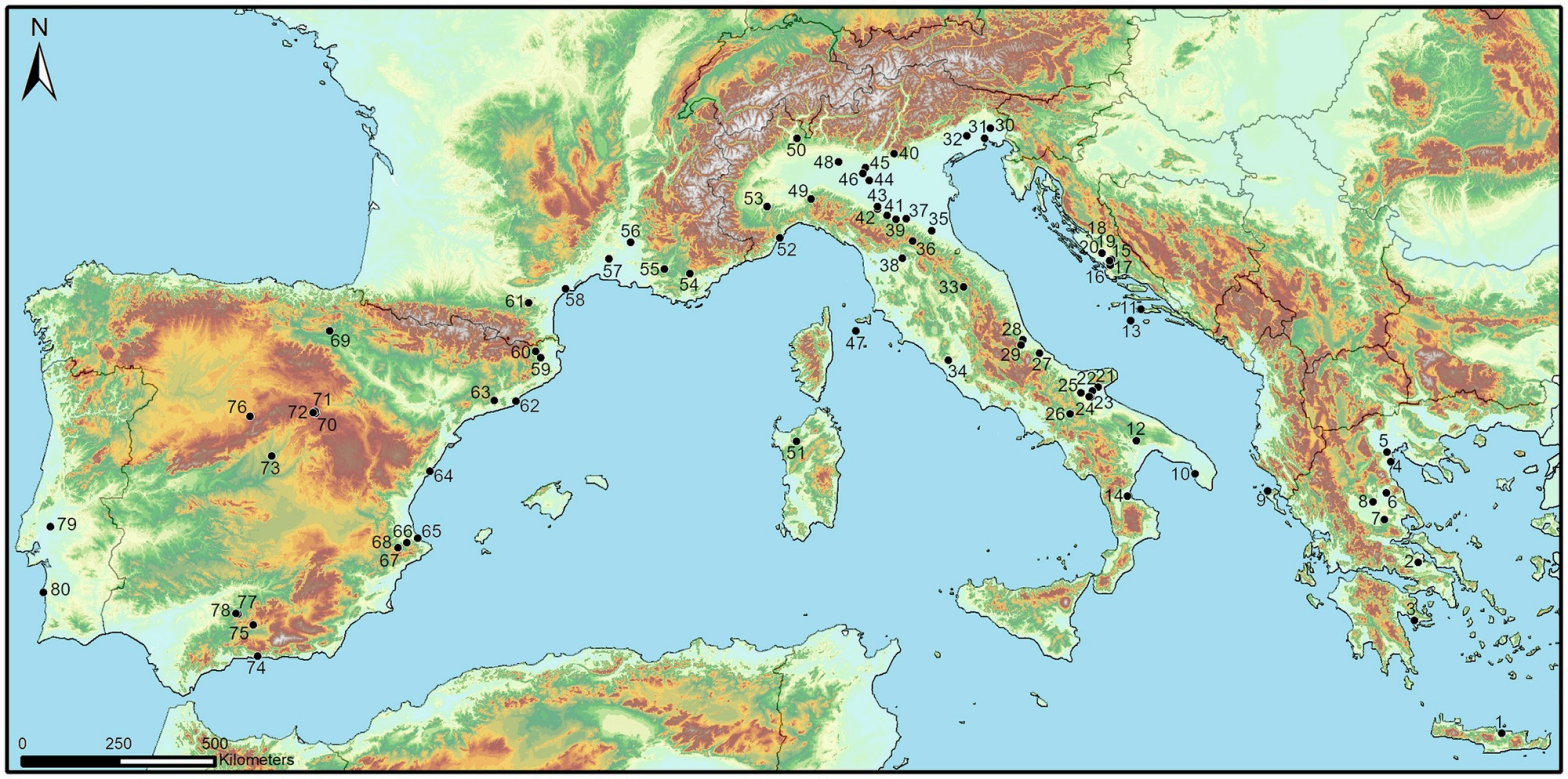
Geographical framework of the study and studied sites. 1) Knossos; 2) Sarakenos; 3) Franchthi; 4) Revenia-Korinou; 5) Paliambela-Kolindros; 6) Rachmani; 7) Achilleion; 8) Platia Magoula Zarkou; 9) Sidari; 10) Torre Sabea; 11) Lokvica; 12) Trasano; 13) Susak; 14) Favella della corte; 15) Pokrovnik; 16) Danilo-Bitinj; 17) Konjevrate; 18) Rasinovac; 19) Krivace; 20) Vrbica; 21) Masseria Candelaro; 22) Passo di Corvo; 23) Masseria Pantano; 24) Ex-Palestra GIL; 25) Masseria Acquasalsa; 26) La Starza; 27) Marcianese; 28) Colle Cera; 29) Catignano; 30) Sammardenchia; 31) Piancada; 32) Fagnigola; 33) San Marco di Gubbio; 34) La Marmotta; 35) Fornace Cappuccini; 36) Cialdino; 37) Casalecchio di Reno; 38) Mileto; 39) Savignano sul Panaro; 40) Lugo di Grezzana; 41) Fiorano Modenese; 42) Rivaltella; 43) Bazzarola; 44) Campo Ceresole; 45) Isorella; 46) Ostiano Dugali; 47) Cala Giovanna; 48) Sergnano; 49) Brignano Frascata; 50) Pizzo di Bodio; 51) Su Coloru; 52) Arene Candide; 53) Alba; 54) Fontbregoua; 55) Mourre de la Barque; 56) Baratin; 57) Mas de Vignoles; 58) Peiro Signado; 59) La Draga; 60) Plansallosa; 61) Jean Cros; 62) San Pau del Camp; 63) Guixeres de Vilobí; 64) Costamar; 65) El Barranquet; 66) Cova de l’Or; 67) Cova Sarsa; 68) Mas d’Is; 69) Los Cascajos; 70) Abrigo de la Dehesa; 71) La Lámpara; 72) Revilla del Campo; 73) Casa Montero; 74) Cueva Nerja; 75) Castillejos de Montefrío; 76) La Vaquera; 77) Murcielagos de Zuheros; 78) Castillo de Doña Mencía; 79) Cortiçois; 80) Vale Pincel I. Map has been prepared using ArcGIS 10.3 using the GTOPO30 global digital elevation model (DEM) with a horizontal grid spacing of 30 arc seconds (Europe and Africa) developed by the U.S. Geological Survey’s EROS Data Centre.

Lithic assemblages from a total of 80 Neolithic sites, corresponding to 92 different occupation phases, were analysed. The study of all lithic assemblages included in this research has been carried out in collaboration of the following institutions: British School at Athens and Knossos Research Centre (Knossos); Ephorate of Antiquities of Boeotia and Archaeological Museum of Thebes (Sarakenos Cave); Ephorate of Antiquities of Argolida and the Archaeological Museum of Nafplion (Franchthi); Ephorate of Antiquities of Pieria (Revenia-Korinou); Ephorate of Antiquities of Thessaloniki Region and University of Thessaloniki (Paliambela-Kolindros); Ephorate of Antiquities and Diachronic Museum of Larissa (Achilleion, Platia Magoula Zarkou and Rachmani); Archaeological Museum of Corfu (Sidari); Università di Pisa (Torre Sabea, Colle Cera, Catignano, Isorella, Cala Giovanna and Sergnano); UMR 5608 TRACES CNRS / Université de Toulouse Jean Jaurès (Trasano, Baratin, Mas de Vignoles, Peiro Signado and Jean Cros); Archäologische Sammlung and Centar Za Kulturu Vela Luka (Susak and Lokvica); Museo delle Civiltà-Museo preistorico etnografico Luigi Pigorini (La Marmotta, Favella della corte); Muzej grada Šibenika (Pokrovnik, Danilo-Bitinj, Konjevrate, Rasinovac, Krivace and Vrbica); Soprintendenza Archeologia, Belle arti e Paesaggio per le province di Barletta-Andria-Trani e Foggia (Passo di Corvo, Masseria Pantano, Ex-Palestra GIL and Masseria Acquasalsa); Museo delle Origini, Università di Roma ‘La Sapienza’ (Masseria Candelaro); Soprintendenza per i beni archeologici di Salerno e Avellino (La Starza); Università degli studi di Siena (Marcianese); Soprintendenza Archeologia, belle arti e paesaggio del Friuli Venezia Giulia and Museo Friulano di Storia Naturale (Sammardenchia and Piancada); Museo del Friuli Occidentale (Fagnigola); Soprintendenza Archeologia Belle Arti e Paesaggio dell’Umbria and Museo Archeologico Nazionale dell’Umbria (San Marco di Gubbio); Soprintendenza Archeologia, belle arti e paesaggio per la città metropolitana di Bologna e le province di Modena, Reggio Emilia e Ferrara (Fornace Cappuccini, Casalecchio di Reno, Savignano sul Panaro, Fiorano Modenese, Rivaltellaa and Bazzarola); Università degli studi di Firenze (Cialdino, Mileto, Pizzo di Bodio and Su Coloru); Università degli studi di Trento (Lugo di Grezzana); Soprintendenza Archeologia, Belle Arti e Paesaggio per le province di Cremona Lodi e Mantova and Museo Archeologico Platina (Campo Ceresole); Soprintendenza Archeologia, Belle Arti e Paesaggio per le Province di Bergamo e Brescia and Museo Civico di Scienze Naturali (Ostiano Dugali); Soprintendenza Archeologia del Piemonte and Musei Reali di Torino (Brignano Frascata and Alba); Soprintendenza Archeologia della Liguria, Museo di Archeologia Ligure and Museo del Finale Ligure (Arene Candide); Musée de préhistoire des gorges du Verdon (Fontbregoua); UMR 7269 Aix-Marseille Université / CNRS (Mourre de la Barque); Museu Comarcal del Banyoles (La Draga); Universitat Autònoma de Barcelona (Plansallosa); Museu de la Ciutat de Barcelona (San Pau del Camp); Universitat de Barcelona (Guixeres de Vilobí); Museo de Castellón (Costamar); Museo Arqueológico de Oliva (El Barranquet); Museo Arqueológico de Valencia (Cova de l’Or and Cova Sarsa); Universitat de Valencia (Mas d’Is); Dirección General de Cultura del Gobierno de Navarra (Los Cascajos); Universidad de Valladolid (Abrigo de la Dehesa, La Lámpara and La Revilla del Campo); Centro de Ciencias históricas y Sociales de Madrid CSIC-CCHS (Casa Montero); Fundación Cueva de Nerja (Cueva Nerja); Universidad de Granada (Castillejos de Montefrío); La Vaquera (Museo Arqueológico de Segovia); Museo Arqueológico Municipal de Zuheros (Murcielagos de Zuheros); Ayuntamiento de Doña Mencía (Castillo de Doña Mencía); Centro de Estudos Arqueológicos do Concelho de Oeiras (Cortiçois); Museu de Arqueologia e Etnografia do Distrito de Setubal (Vale Pincel I). These institutions, where the artefacts are conserved and stored, provided us the legal permission to study the archaeological collections, which are publicly accessible to researchers. The geographic area of study extends from the Aegean Sea to the westernmost coasts of Portugal. Chronology spans between ca. 7000 and 5000 cal BCE (S1 Table; Fig 1).

Assemblages were thoroughly examined in order to identify their technological features according to the concept of chaîne opératoire, debitage economy, and raw material economy [63,64]. The selection of ‘glossy tools’ (i.e. tools used for harvesting cereals) was carried out by stereoscopic examination (5× to 60× magnification). At such magnifications all types of glossy surfaces, even the most marginal ones can be correctly identified. Afterwards, glossy tools were analysed through a reflected-light microscope (N300 Nikon Labophot, 50× to 400× magnification) in order to highlight the microtextural characteristics of the use-wear. Raw-material variability has also been carefully considered when analysing use-wear traces and the types of raw-materials are detailed in S1 Table. While the variability between fine-grained varieties is not important enough to affect use-wear micropolish appearance [65], coarser-grained cherts affect use-wear formation and development. Nevertheless, the use of coarser raw materials for insert production remains anecdotal in the studied area/period. Obsidian is different in that, contrarily to chert, obsidian inserts first develop a matt polish and the characteristic cereal-gloss is formed on the edge only later.

A ^14^C dataset was built associated with the analysed contexts, including 543 radiocarbon dates from 72 sites (8 of the analysed sites do not have any radiocarbon dates available) of which 231 are on short-lived samples (S1 File). Radiocarbon dates obtained for the same archaeological context and corresponding to the same depositional event, such as two fragments of charcoal from the same stratigraphic unit, were tested following the T-test (S2 File). When the test was positive, the uncalibrated dates were combined using the tool R_Combine of the program OxCal 4.3, then their pooled mean was calibrated [66]. Kernel Density analysis (KDE) [67,68,69] is used to represent the temporal distribution of diagonally-, parallel-hafted and oblique sickle blades in the central and western Mediterranean area. The OxCal 4.3 tool KDE_Plot that provides a kernel density distribution for the samples [70] is implemented with collected data. Compared to the Summed Calibrated Probability Distribution (SPCD), Kernel Density Plots have the advantage of removing the high frequency noise of the SCPDs, retaining only the lower frequency signal and thus eliminating data dispersion.

Collected ^14^C dates were also statistically analysed using the OxCal 4.3 chronological models [66]. Radiocarbon measurements were constrained in a model formed by four phases organized in four sequences, each one defined by a Boundary Start and a Boundary End and including dates for a specific sickle blade type. Only dates obtained on short-lived samples were selected in order to discard the possibility of an old-wood effect for the first occurrence of each harvesting tool (S3 File). Data modelling through OxCal 4.3 software was used to calculate the time span of the boundaries which describe the introduction of each sickle blade type and provide this information according to 1σ and 2σ probabilities. To measure the time span between the first occurrence of diagonally-hafted sickle blades and the introduction of parallel-hafted sickle blades, the OxCal 4.3 Difference tool was used. Using this function the duration of this interval was calculated taking into account the two Boundary Starts of the analysed sequences introduced in the model.

## 3. Results

### 3.1 Going westward: The Aegean Sea and mainland Greece

The Neolithic of south-eastern Europe, and especially of the Aegean area and mainland Greece, is traditionally believed to represent the first diaspora of farming populations. Here, the Neolithic way of life was fully exported, comprising a set of domesticated plants and animals, mud brick architecture, grinding-stone and pottery technology, as well as symbolic and aesthetic expressions [11,71]. The Aegean was first occupied by farmers during the first half of the 7th millennium BCE, as exemplified by the recent radiocarbon dating of the Aceramic or Initial Neolithic from Knossos Layer X (6800–6600 cal BCE) [72]. The site testifies the onset of farming practices in Crete, with domesticated animals (goats, sheep, pigs, cattle, and dogs), domesticated cereals (*Triticum sp*., *T*. *aestivum/durum*) and legumes (*Pisum sp*.) [73].

The flaked stone assemblage recovered from the 1997 excavation of the Central Court in the Palace of Knossos has been fully analysed in the frame of this research (S1 Table), offering the earliest evidence of the use of harvesting tools in the Aegean. The assemblage had been the object of previous studies by J. Conolly and, later, by M. Kaczanowska and J. Kozlowski, resulting in quite different readings. While Conolly [74] tends to interpret the collection as results of technical choices adopted by a small, rather isolated, farming community, Kaczanowska and Kozlowski [26] consider the assemblage to show typical aspects of the Aegean Mesolithic stone tool tradition, suggesting continuity between pre-Neolithic seafaring groups and the first settlers on Crete. The main trait that would associate the Layer X assemblage to the Aegean Mesolithic is the flake-based technology and the presence of specific typologies of backed and notched/denticulated tools. Kaczanowska and Kozlowski also indicate the absence of sickle inserts as one of the features of the Mesolithic tradition. Two specimens (one bladelet and one retouched flake) showing the characteristic gloss from harvesting cereal were indeed considered intrusive by the authors. However, such an interpretation is questionable, being exclusively based on technological and typological criteria, and not taking into account the stratigraphic position of the finds.

**Fig 2:**
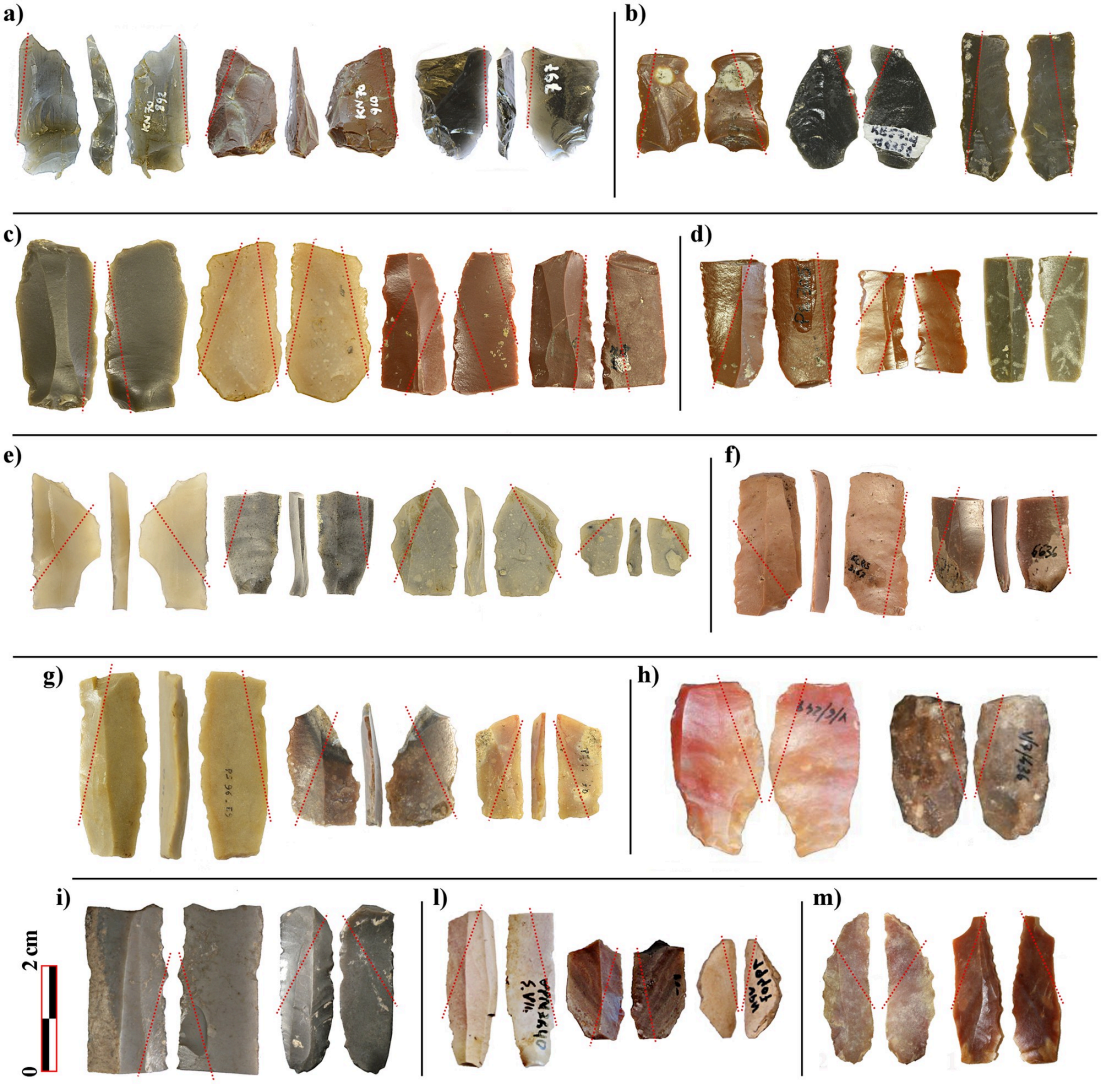
Diagonally-hafted inserts. Crete- a) Knossos; Greece- b) Paliambella; c) Achilleion; d) Revenia; Italy- e) La Marmotta; f) Fornace Cappuccini; France- g) Peiro Signado; Spain- h) Guixeres de Vilobí; i) Castillejos de Montefrío; Portugal- l) Vale Pincel I; m) Cortiçois. The red dots indicate the distribution of the glossy area. All photos have been realised by the authors (NM and JFG).

The use-wear analysis carried out has added further data to the discussion, highlighting the presence of six more glossed inserts (Fig 2a).

**Fig 3:**
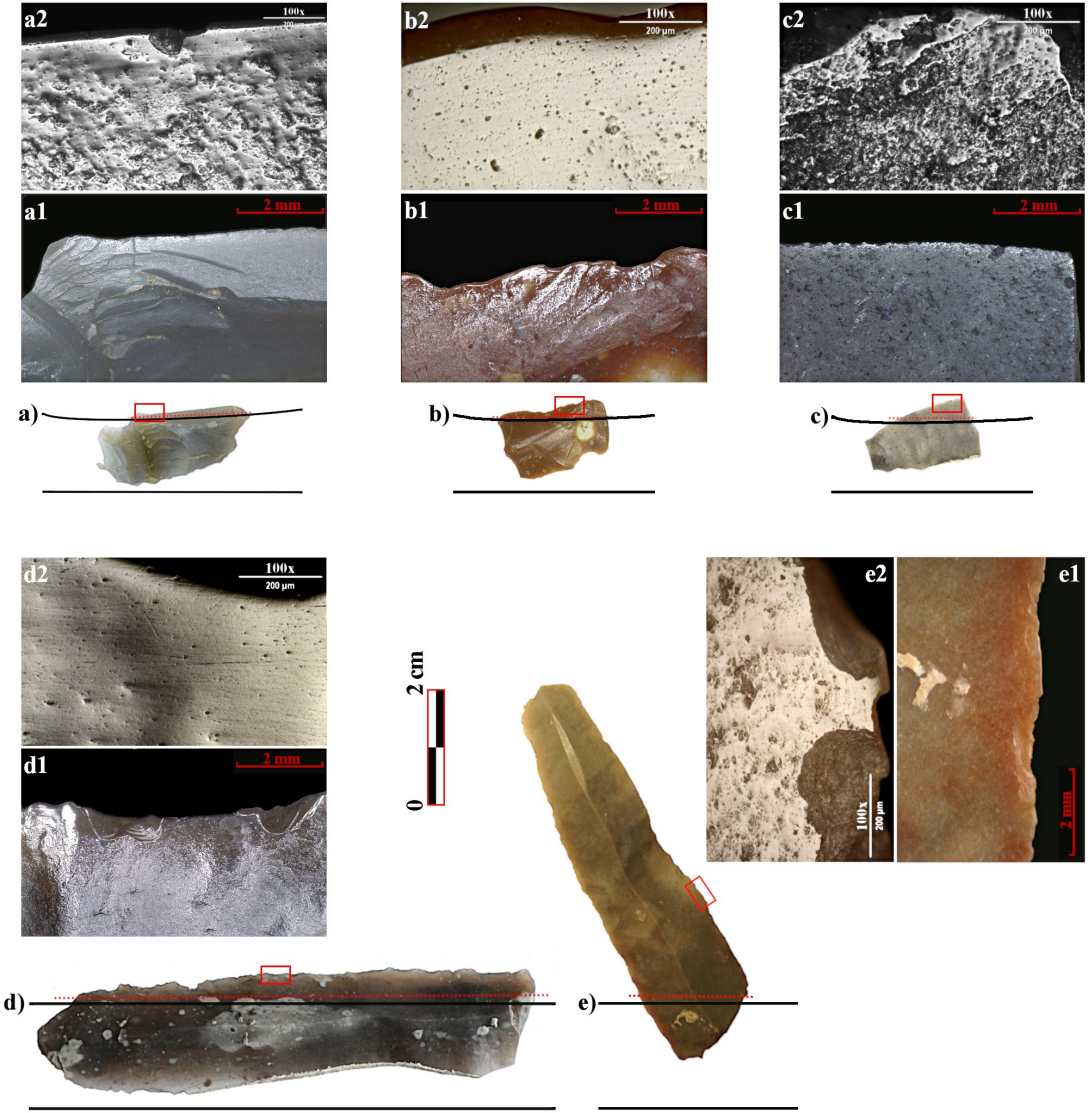
Macro and micro- use-wear from different sites and insert-types. a) Knossos, a1- macrowear, marginal gloss, 10x; a2- microwear, smooth, marginal, cereal polish, 100x. b) Paliambella, b1- macrowear, well-developed diagonal gloss, 10x; b2- microwear, well-developed, pitted and striated cereal gloss. c) La Marmotta, c1- macrowear, marginal gloss, 10x; c2- microwear, smooth, marginal, cereal polish, 100x. d) Danilo, d1- macro, well-developed parallel gloss, 10x; d2- flat, well-developed, pitted and striated cereal polish, 100x. e) Revilla del Campo, e1-macro, rounded edge, 10x; e2- flat, well-developed cereal polish, 100x. All photos and micrographs have been realised by the authors (NM and JFG).

They are made on both Melian obsidian and radiolarite. Except for the one bladelet already mentioned, inserts are made on flakes. Four of the flakes have been retouched laterally, likely to reduce the blank width. Inserts show quite a marginal gloss, faintly diagonal or parallel to the edge (Fig 3a).

They were presumably hafted to form a slightly serrated edge, within a composite sickle. Use-wear patterns indicate the harvesting of cereals; despite the reduced sample, certain variability has been observed in the wear pattern, probably because of the harvesting of plants in different states of ripeness [38]. Harvesting crops before full ripeness is well-known behaviour among traditional Mediterranean farming communities and might respond to a risk-reduction strategy, to reduce grain loss, a response to food shortages, but as well to specific food habits [60,62].

The use of both backed retouched and unretouched blanks as sickle inserts is common in many other 8th and 7th millennia Neolithic collections. The site of Ulucak VI [75] in Aegean Turkey or, PPNB and Ceramic assemblages in Cyprus, like Shillourokambos (phases B-C) [41], Khirokitia [39] and Paralimni-Nissia [76] can be cited. Blades are more frequently used, but flakes are also used, especially in industries characterised by low technical investment and a relative abundance of flake blanks (i.e. Khirokitia). Knossos might represent a similar case. We are inclined to agree with Perlès [77], when she suggests that the expedient methods implemented for lithic reduction at Knossos were due to the absence of specialist knappers mastering the pressure flaking technique among the small group of pioneer farmers. Following this perspective, Knossos Layer X assemblage would represent a good example of the adaptation process that Neolithic groups experienced during their spread, due to the changing material conditions and to the ‘selective’ nature of migration [78] resulting in a diverse social composition of the migrant group with respect to the original society. It is remarkable that the use of bipolar-on-anvil technique is probably the most widely-diffused and easiest method for obsidian reduction, adopted in different geographical and chronological contexts. In this sense, several insular contexts where bipolar-on-anvil technique is used for obsidian flaking may be cited, such as the Early Neolithic of Corse [79], in the Sardinian Middle and Late Neolithic and Chalcolithic [80], and even during the historical period in the Canary Islands (5th–14th centuries CE) [81]. The technical choices adopted by the first settlers at Knossos might not be a direct expression of cultural identity, but an adaptation to specific social and environmental conditions [74].

Farming communities arrive in mainland Greece one or two centuries later, around 6600 cal BCE. Thessaly and, in lesser extent, Aegean Macedonia are the areas characterised by a denser presence of Neolithic occupations, with an estimated number of over 250 Early Neolithic sites [71]. The first farming communities occupied permanent and long-lived villages, primarily subsisting on cereal (e.g. *Triticum* sp., *Hordeum* sp.) and pulse crops (e.g. *Lens*, *Pisum*) [82]. This area, characterised by semi-arid Mediterranean climate conditions, probably represented an ideal place for crop cultivation, an environment close to the conditions in which cereals were first domesticated in the Near East [31].

Early Neolithic assemblages have been analysed from the sites of Revenia and Paliambella (Greek Macedonia), Achilleion (Thessaly), and Franchthi (Peloponnese) (S1 Table). Flaked stone assemblages are here characterised by a blade-oriented technology, using a diversity of local, regional and supra-regional raw materials, comprising cherts and radiolarites of different qualities, quartz, chalcedony, and Melian obsidian. Raw material availability is uneven across Greece and different circulation networks probably existed from early phases of the Neolithic. Maritime routes of Melian obsidian circulation expanded towards mainland Greece and Anatolian coasts, while terrestrial distribution routes probably involved other high-quality rocks such as honey-flint [71]. Glossy blades are one of the most representative classes of tools in the Greek Early Neolithic. At Achilleion and Revenia glossy pieces are represented by high percentages, between 17–18% of the sample (including both retouched and unretouched flaked blanks), while at Paliambella inserts represent 11% of the analysed assemblage. Inserts are larger in size than the harvesting tools found at Knossos. This can be related to the availability of a broader array of raw materials and to more skilled production, allowing wider and longer blanks to be obtained.

Glossy tools were mostly produced by fragmenting blades; the resulting fragments were then used as inserts within composite harvesting tools, to form a slightly serrated edge, as already observed at Knossos (Fig 2b–2d). Blanks show little modification. Edges are generally unretouched; only flakes are occasionally backed in order to reduce their width. A marginal retouch of the active edge to prolong its effectiveness is fairly common, but it is not systematically carried out, suggesting that, once exhausted, the active edge was repaired by replacing the insert with a new one, or, to a lesser extent, by using another edge of the same item, as about the 20% of the specimens show gloss on both edges. At a few sites, harvesting inserts made on longer and wider blade blades are also documented. Two implements at Revenia and four implements at Franchthi fit within this category. Blanks are unfortunately fragmentary and their interpretation remains ambiguous, so it is not possible to estimate the original size of the insert. The lustre runs parallel to the edge, forming a straight cutting edge. This type of hafting mode might belong to a different type of harvesting tool. Nevertheless, no relevant differences in polish texture have been noted between the two insert-types.

All inserts display a well-developed gloss, suggesting a prolonged use for harvesting practices (Fig 3b). Traces present the typical flat and striated polish obtained from the harvesting of ripe cereals for dozens of hours. The strong development of the wear, together with the high percentages of glossy implements within Thessalian and Macedonian lithic assemblages, suggests that agriculture was a major economic activity, one of the main occupations in which chipped stone tools were used. This data fit well with the results from bioarchaeological analysis, indicating a crop-based diet and great dependence on grain crops [82].

### 3.2. One step forward: The spread of farming in the central and western Mediterranean

After a delay of about 500 years from the initial arrival of farming groups in the Aegean, the Neolithic started to spread towards the Central and Western Mediterranean (CW), between *ca*. 6000 and 5300 cal BCE. This process has been described as an arrhythmic expansion characterised by sudden diffusion followed by breaks and further rapid movements [22,83]. The first areas to be colonised were the Adriatic coasts of Albania, Montenegro, Croatia, and South Italy. A rapid, maritime, diffusion further west then took place just a century or two later, reaching the Gulf of Lyon already around 5840–5740 cal BCE (i.e. Peiro Signado) and the NE of the Iberian Peninsula, around 5630–5550 cal BCE (i.e. Guixeres de Vilobí). Maritime travel allowed the establishment of new settlements and the maintenance of contact between them, as also through long-distance mobility as recently demonstrated [84]. In the early stages, the expansion is associated with groups bearing Impressed Ware pottery, later diverging in regional entities through mechanisms that are not fully understood at present [85,86].

Seafaring colonists cultivated a large diversity of cereals, pulses and oil plants although some remarkable difference can be noted. Pulses are much less represented in the Western Mediterranean than in the Balkan area [32,87]. In addition, regional preferences can be seen in the type of cultivated cereals. While the glume type prevails in Italy and Dalmatia [88,89], free-threshing wheat appears dominant on the eastern and southern façade of the Iberian Peninsula [90]. Even if such differences are still little understood, they might well indicate a regional adaptation of the farming package.

**Fig 4:**
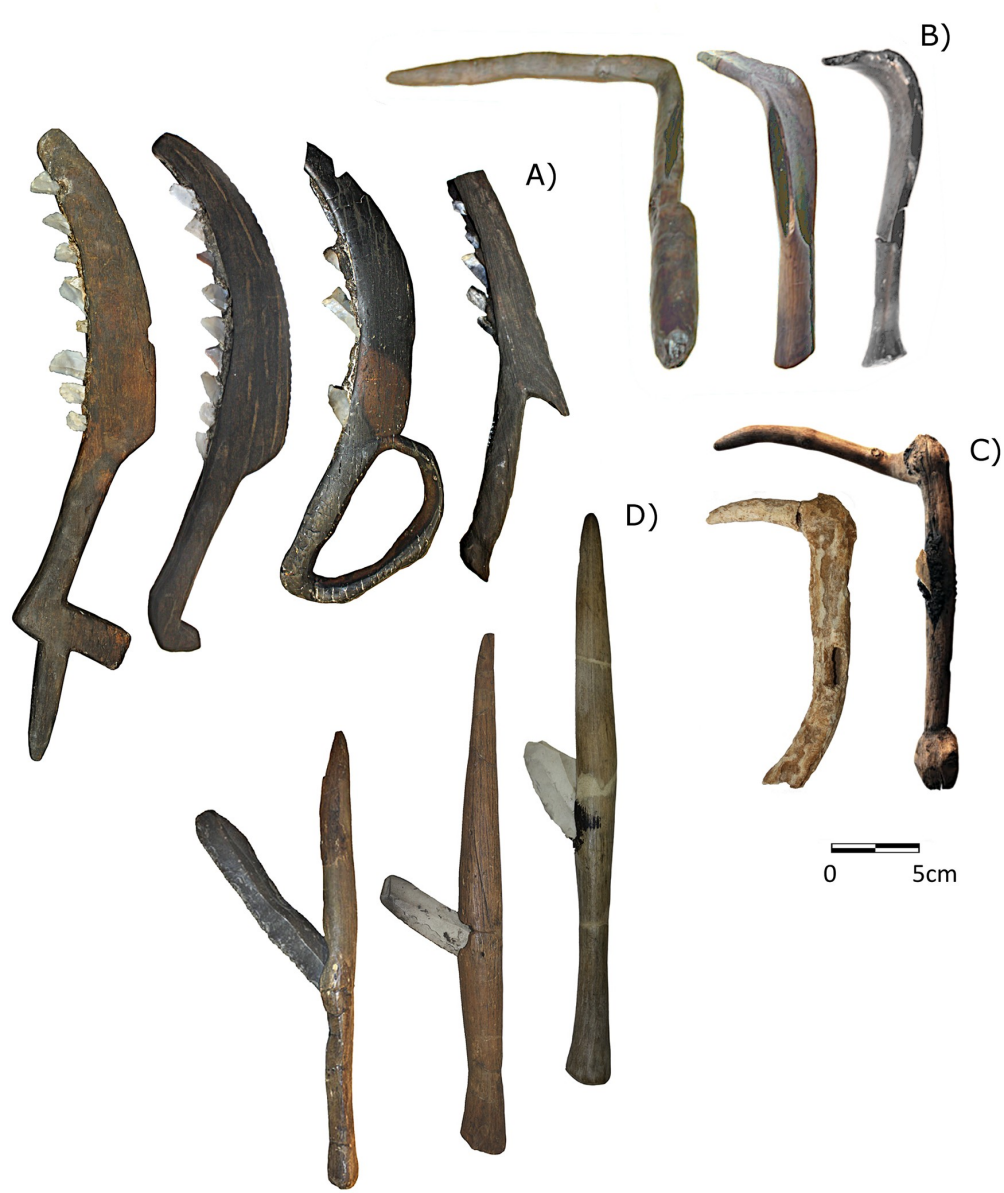
Complete sickles from the Central and Western Mediterranean. A) Wooden sickles from La Marmotta (Italy) (Rome, Italy); B) Reaping knives from La Draga (Girona) [107]; C) Reaping knives from La Draga (wood) and from Costamar (antler) [108]; D) Reaping knives from Egolzwil 3 [109]. Photos of La Marmotta, Costamar and Egolzwil 3 sickles have been realised by the authors (NM and JFG). Photos of La Draga have been modified from ref. [107]. This figure is similar but not identical to the original image and is therefore for illustrative purposes only.

The harvesting technologies of the first farmers in the Central and Western Mediterranean are well documented by a large number of sites (Fig 1). Our data is particularly detailed thanks to submerged, lakeshore sites, like La Marmotta in Central Italy, where several fully-conserved wooden sickles have been recovered. Sickles were made of a curved or slightly curved handle in which lithic inserts were hafted more or less diagonally, so forming a coarsely serrated edge (Fig 4).

The sickles were between 22 and 31 cm long, with a cutting edge measuring on average 14 cm, supporting the idea of tools adapted for harvesting a relatively small number of cereal plants [57]. This hypothesis is further supported by the analysis of the glossy tools: harvesting inserts show more marginal use-wear traces compared with Greek specimens, suggesting a less intensive utilisation of the tool (Fig 3c). In addition, considering the flaked stone assemblages from the early ‘pioneer’ settlements in the CW Mediterranean, the reduction in the number of inserts with regards to the Southern Balkan collections is striking. Glossy tools represents less than the 4% of the analysed assemblage (S1 Table) at Torre Sabea, La Marmotta, Pokrovnik, Arene Candide, Fornace Cappuccini, Pendimoun, Peiro Signado, Les Guixeres de Vilobí, El Barranquet and Murciélagos de Zuheros, just to cite a few of the most representative sites. Despite the fact that the functional status of the site (e.g., open-air village, logistic settlement, sheepfold cave, etc.) might influence the representation of harvesting inserts within the assemblage, this pattern is fairly constant across the entire CW Mediterranean.

In most of the above-mentioned sites, the production of harvesting inserts is characterised by low technical investment. Several sources of good-quality cherts are available in the Central and Western Mediterranean: Gargano formations in South Italy, Umbro-Marchigian Scaglia Rossa in Central Italy, Lessini chert in North Italy, Bedoulian chert in Southern France, Ebro Basin and Ulldemolins cherts in north-eastern Iberia (see [91] and references therein). However, the circulation of those materials is uneven during the first phases of Neolithic expansion and cherts of local or regional origin were also used for harvesting tool production. For example, at La Marmotta, Peiro Signado, or Guixeres de Vilobí, some of the earliest and most representative Neolithic villages in the CW Mediterranean, local and regional raw materials dominate the assemblages. Inserts are produced by breaking small blades or bladelets knapped on-site by hand pressure or indirect percussion flaking technique and, to a lesser extent, by using small flakes (Fig 2e–2m). It is highly possible that the harvesters were capable of producing the inserts themselves when necessary, the technology involved in their production being rather unspecialised. Adaptability and reliability were probably important criteria for exporting harvesting technology into new and still little-known environments. Neolithic pioneer groups initially occupied unexplored and scarcely inhabited territories and, therefore, they were in need of a reliable and adaptable harvesting tool, not too demanding in terms of technological investment and raw material quality for insert production.

Curved sickles were used for either mid- or low-height harvesting; the presence of abrasive traces on some inserts suggests that grain was cut halfway down the straw or indeed, fairly close to the ground. Not only the ear, but part of the straw was therefore collected. The usage of the straw for different craftwork (baskets, cloths, daub-making, etc.) is documented at several Neolithic sites in the CW Mediterranean [56,92].

It can be stated that, throughout the migration process, harvesting technologies were adapted to different economic, technological and social conditions. The difference between mainland Greece and the rest of CW Mediterranean in insert frequency and in use-wear polish development can be explained as the result of a smaller scale of production. In addition, given the larger average size and number of lithics, one might suppose that the overall size of the tool was larger in Greece (thicker and longer sickles) than the Impressa Ware sickles. Unfortunately, no complete sickles have been recovered so far in Greece, hindering direct comparison. Karanovo-type inserts from Bulgaria show some similarities with the Greek specimens. In the Bulgarian Early Neolithic, inserts display an angular shiny gloss, from slightly oblique to diagonal. Sickle blades with parallel lateral polish are rare. There is little typological standardisation in insert morphology; both unretouched and variably retouched blades–more rarely flakes–were used [93]. Inserts were hafted in curved antler handles, with a maximum length of between 30 and 38 cm, with a cutting edge averaging 18 cm [94]. Nevertheless, a more detailed study, combining both archaeological and experimental data, is needed to carry out a comparison with La Marmotta sickles.

### 3.3. Changing technologies: Harvesting tool evolution in the seventh-fifth millennia BCE

Despite variations in insert recurrence, size and utilisation rate, the technology on which the first harvesting tools are based is the same at a Mediterranean scale: a haft in which stone tools are placed to form a more or less coarsely serrated edge. Inserts are used until exhaustion and then replaced with new ones, largely following the scheme of a ‘maintainable technology’ [95]. This model, however, does not represent the only available choice. Since at least the 10-9^th^ millennia cal BCE, different types of harvesting inserts appear in the archaeological record in the eastern Mediterranean.

In mainland Greece, harvesting tools made on larger blanks are occasionally documented-always in coexistence with smaller implements-from the earliest Neolithic phases. A few of them have been noted at Revenia, Franchthi [96] and Argissa [71], with chronologies spanning approximately between 6450 and 5500 cal BCE. Such blades were mainly flaked by pressure techniques (with a shoulder or a chest crutch, or with a lever system [97]). Instead of being split into fragments and hafted to form a coarsely serrated cutting edge, inserts were hafted to form a straight cutting edge, parallel to the handle of the harvesting tool.

**Fig 5:**
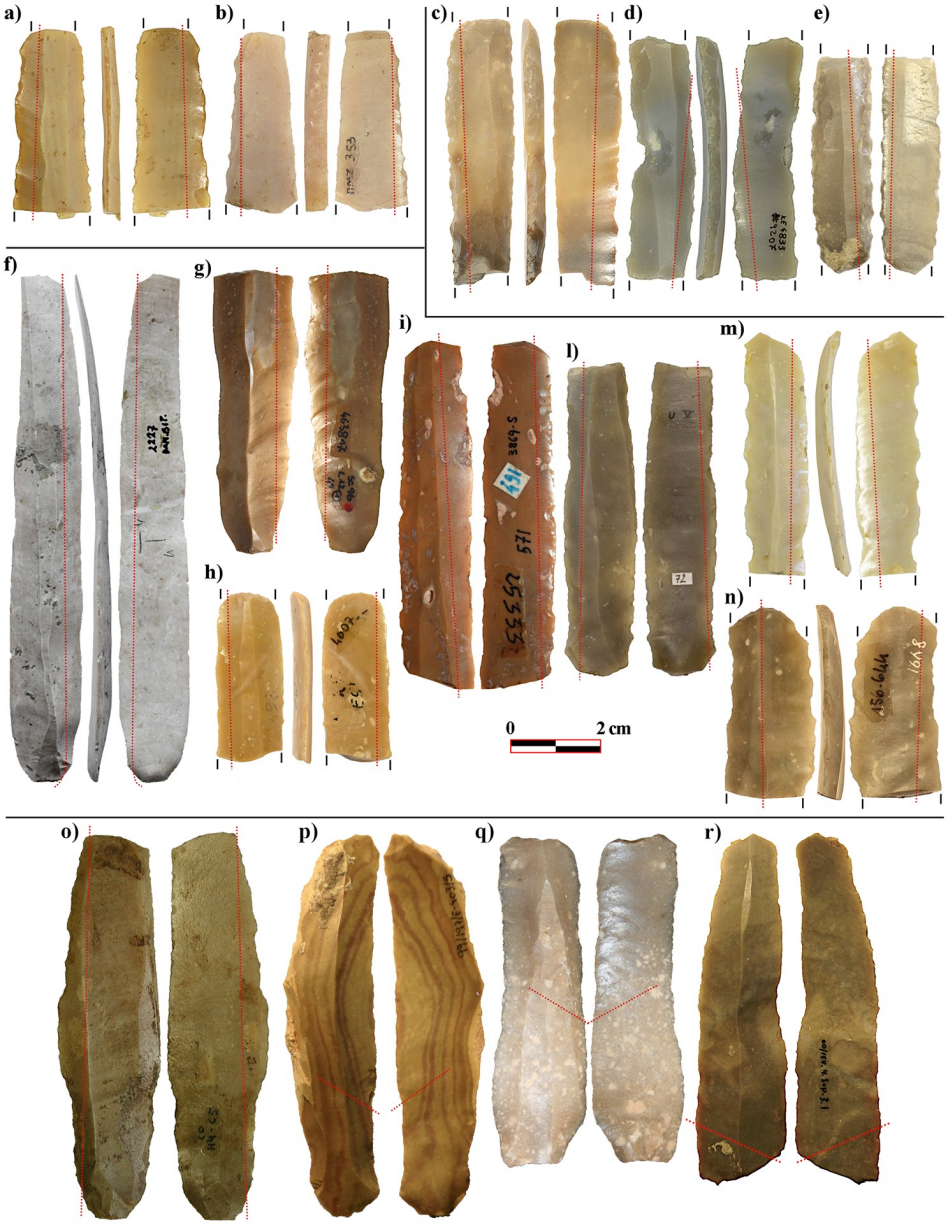
Parallel-hafted inserts. Greece- a) Achilleion; b) Platia Magoula Zarkou; c) Sarakenos Cave; d) Paliambella; e) Rakhmani; Croatia- f) Danilo; Italy- g) Sammardenchia; h) Fagnigola; i) Arene Candide; l) Rivaltella-Ca’ Romensini; m) Savignano sul Panaro; n) Fiorano; Spain- o) Cova de la Sarsa; p) La Lámpara; q) Casa Montero; r) La Revilla del Campo. The red dots indicate the distribution of the glossy area. Broken inserts are indicated by black lines. All photos have been realised by the authors (NM and JFG).

Glossy tools on long blades start to be increasingly common from the late Early and Middle Neolithic (Fig 5).

They are well attested in Thessaly, in Achilleion phases III and IV, between 6240–5480 cal BCE while at Franchthi, in Argolis, they become the dominant type in the Middle Neolithic layers (ca. 5900–5600 cal BCE) [97]. At Platia Magoula Zarkou, in the western Thessalian plain, parallel-hafted inserts are well documented from the Middle and early Late Neolithic, around 5850–5450 cal BCE. In both sites, larger blades hafted in parallel fashion coexist with shorter inserts, suggesting that different types of harvesting tools were used at the same time. However, the use of large and wider blades would become predominant, at least at some sites, also during Late and Final Neolithic phases. This is the case of Sarakenos Cave in Boeotia (*ca*. 5200–4250 cal BCE), Paliambella in Greek Macedonia (*ca*. 5450–4350 cal BCE) [98], and Rachmani in Thessaly (*ca*. 4700–4300 cal BCE).

Parallel-hafted inserts may have appeared as early as ca. 5800–5500 cal BCE on the Dalmatian coasts (Fig 3d). At Crno Vrilo inserts of this type are documented between 5900–5300 cal BCE-even if this information is based on a techno-typological study by Korona [99] and should be revised. Nevertheless, both typologies of inserts coexist at this site; the use of parallel-hafted inserts would indeed be widespread in the area only starting from *ca*. 5300 cal BCE, with a complete substitution of diagonally hafted inserts during Danilo-pottery occupation phases (i.e. Danilo-Bitinj, Vrbica, Krivače, Pokrovnik). A phase of coexistence of both types of harvesting tools between *ca*. 5800/5600 and 5600/5400 seems plausible on the basis of current data, despite evidence still being fragmentary [57].

In North Italy the earliest evidence of parallel hafted blades is known from the north-eastern sector of the peninsula, at Sammardenchia and Fagnigola, around 5600–5400 cal BCE. Nevertheless, no short-lived samples have been published for this area, and the current chronology may be too high. Parallel inserts would persist for almost one millennium, until approximately 4500 cal BCE, as attested at Sammardenchia and Piancada [100]. Diagonally-hafted implements are not known at these sites.

Data from the Po Plain and the Liguria Coast suggest instead a nuanced change in the harvesting toolkit. Diagonally-hafted inserts are well documented between *ca*. 5300 and 5000 cal BCE in sites of the Fiorano (Bazzarola, Lugo di Grezzana) and Vhò cultures (Alba, Brignano Frascata, Campo Ceresole, Dugali Alti). Nevertheless, contemporary sites (i.e. Fiorano Modenese, Casalecchio di Reno, Isorella and Sergnano) between *ca*. 5500 and 4700 cal BCE, in the same geographical and cultural areas are characterised by inserts on longer and wider blades, hafted in parallel. Fiorano Modenese has provided the earliest date (5610–5470 cal BCE); but it is an old pre-AMS date that should be considered cautiously [101]. ^14^C dates from Casalecchio di Reno and Isorella delay the adoption of parallel hafted blades in the Po Plain for a few centuries, respectively 5330–5220 cal BCE (charcoal) and 5210–5070 cal BCE (charred caryopsis). In addition, at some sites such as Brignano Frascata and Savignano sul Panaro (5470–5210 cal BCE), both typologies seem to coexist. Parallel-hafted blades would definitely become predominant during the successive Square Mouth Pottery (SMP) period, between *ca*. 5000 and 4350 cal BCE, as documented at Rivaltella Ca’ Romensini, Arene Candide SMP-layers [102], Quinzano Veronese and Travo [103].

In Southern France, parallel inserts are known from several sites dated between 5400 and 5300 cal BCE, such as the open-air site of Le Baratin, and the caves of Fontbregoua and Pendimoun. As in Italy, parallel inserts replace the pre-existent harvesting technology based on small diagonally-hafted inserts [53]. At Le Baratin, Fontbregoua and Pendimoun (cardial layers) [104] both type of inserts probably coexisted, despite being very fragmentary, suggesting a nuanced transition between the two types, as already observed in North Italy. Finally, in the Iberian Peninsula harvesting tools bearing whole, parallel-hafted blades first appear after 5300 cal BCE, in both coastal (e.g. Sant Pau del Camp, La Draga) [105] and inland areas (e.g. Cueva Chaves–[106], La Vaquera, La Revilla del Campo). At Los Cascajos settlement, in Navarre, a gradual replacement of diagonally-hafted inserts in favour of larger parallel-hafted blades has equally been documented [55].

In the North-East of the Iberian Peninsula, a third type of harvesting tool has been identified. Its identification has been possible through finds at the lake-dwelling site of La Draga (Catalonia) [107]; an identical tool, made on antler, has also been recovered from the site of Costamar, in Castellón [108] (Fig 4). These are L-shaped sickles formed by a straight shaft, a transversal branch, and a long blade inserted obliquely to the straight shaft. The position of the blade, obliquely-placed and not parallel to the haft, represents the main variation from the other reaping tools found at La Draga (Fig 3e). Nevertheless, while tools bearing parallel-hafted blades have been documented in almost the whole north-western Mediterranean Arc, this latter variant has until now been documented exclusively in a few sites in central Spain (La Lámpara, La Revilla del Campo and Casa Montero), at the above-mentioned sites on the north-eastern Iberian coast and probably in a few sites in Southern France (Grotte Lombard, Petites Bâties, Basi). Tools with similar features would appear in the late fifth millennium Swiss Neolithic [109] (Fig 4).

Finally, in the Neolithic of the Cantabrian coast and the French Jura, dated to the late fifth millennium cal BCE, the absence of glossy blades in the flaked stone assemblages (sites of Kobaederra, Arenaza, Chalain 3, la Motte aux Magnins) suggests that alternative methods were used for harvesting cereals [110]. Ear plucking, plant uprooting, and reaping sticks [111] could have been well-adapted techniques for cereal harvesting, considering the humid environmental conditions of these regions and the small size of the cultivated fields, as estimated by archaeobotanical analysis [92].

## 4. Discussion

The migration of Neolithic groups across the Mediterranean represents a period of intense cultural transformations, including processes of inheritance, selectivity, drift, isolation-by-distance, acculturation, transfer of ideas and symbols, and the establishment of both short- and long-range interaction networks [112–115]. The spread and adoption of harvesting technology can therefore be viewed within a larger process of change. Harvesting tools are only one component of a larger material culture, the Neolithic package [116], whose components change at different rates and time frames [117,118]. For example, the analysis of the stylistic variation in pottery decorative techniques has allowed the expansion of groups bearing different traditions to be traced across the Mediterranean Basin and their fragmentation in regional entities [119]. Nevertheless, changes in pottery decorative techniques do not correlate with changes in other records, such as harvesting tools. Based on current data, there is no synchronism between stylistic change in pottery and harvesting toolkit composition, and it seems hard to defend the transmission or inheritance of a Neolithic package as a whole.

**Fig 6:**
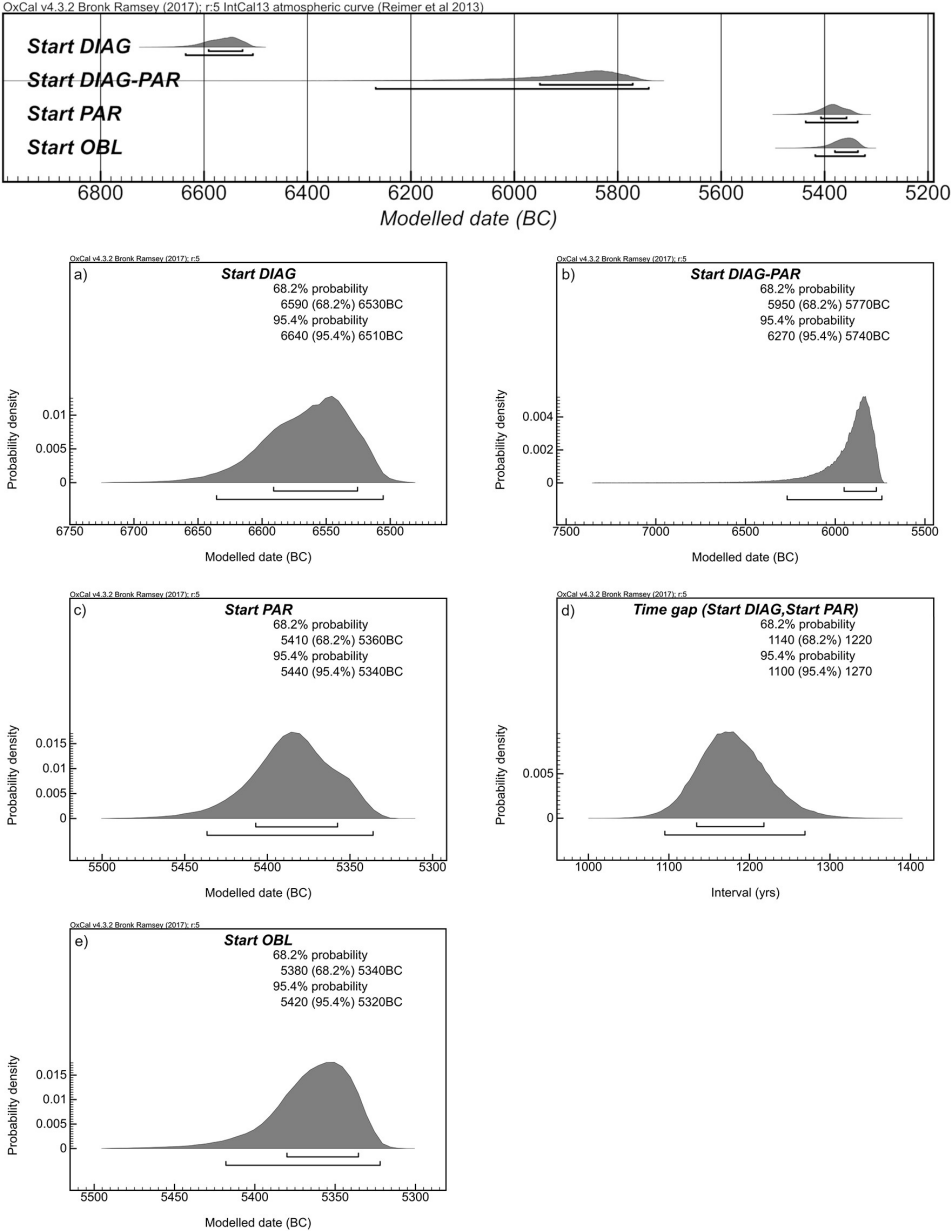
Results of the OxCal modelling for archaeological contexts in the central and western Mediterranean showing the time spans for the introduction of: (a) diagonally-hafted inserts, (b) diagonally- and parallel-hafted inserts, (c) parallel-hafted inserts, (e) single oblique-hafted inserts, and (d) the time span between the introduction of diagonally-hafted inserts and parallel-hafted inserts.

During pioneer phases of the Neolithic expansion, a preference was made towards the most reliable and less demanding harvesting technology. Serrated sickles were spread by seafaring communities bearing different pottery styles. As shown by radiocarbon dates, the introduction of diagonal inserts in the Aegean can be placed between *ca*. 6640 and 6510 cal BCE (Fig 6a).

**Fig 7:**
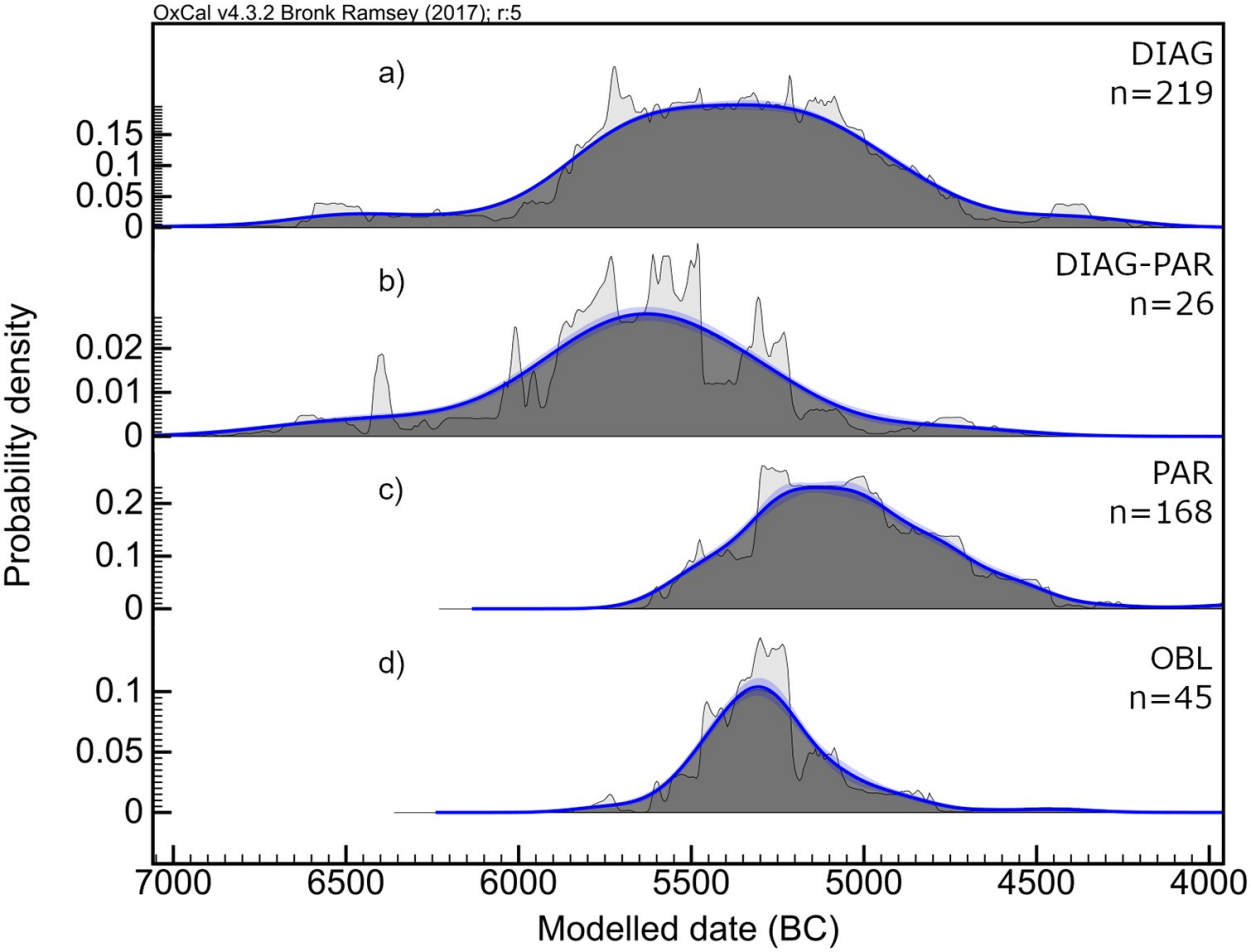
KDE plots showing the temporal distribution of archaeological contexts with presence of: (a) diagonally-hafted inserts, (b) diagonally- and parallel-hafted inserts, (c) parallel-hafted inserts and (d) single oblique-hafted inserts.

From this region, the harvesting technologies would have expanded to the west and north-west, reaching first Southern Italy and the Dalmatian coast at the beginning of the sixth millennium, and rapidly spreading through the Gulf of Lion and the Levantine façade of Iberian Peninsula (Fig 7a).

This pattern is in good agreement with the classic hypothesis of the maritime diffusion of small pioneer groups along the Central and Western Mediterranean coasts [120]. In inland territories, the adoption of this specific kind of tool took place approximately 600/700 years later in the Po Valley, in North Italy, as well as in Andalusia, in southern Spain. It is significant that parallel-hafted blades are occasionally present among these early Neolithic assemblages, between *ca*. 6270 and 5740 cal BCE (Fig 6b). By including 14C dates on charcoal, the appearance of parallel-hafted blades can be taken back a few centuries, to ca. 6450 cal BCE (Fig 7b). The most important point is that, despite the limited number of parallel-hafted specimens and blank fragmentation often making their interpretation difficult, it seems that a diversity of harvesting tools existed from the beginning of the expansion phenomena in the Eastern and Central Mediterranean. This is possibly the case of a few sites in Argolis (e.g. Franchthi), in South Italy (e.g. Trasano, Favella della Corte, La Starza and Masseria Candelaro) and in the Valencia Region (e.g. Cova Sarsa) in which a few parallel-hafted inserts are present. However, they were used much less than the smaller diagonally-hafted inserts. At a microscopic view no clear difference in the use-wear is detectable, both showing the typical cereal polish, indicating that both tool-types were employed for the same activity.

A major but gradual change in the harvesting toolkit took place in a period between *ca*. 5440 and 5340 cal BCE (Fig 6c), with a gap of about 1200–1000 years from the first adoption of diagonal inserts (Fig 6d), over a vast area roughly corresponding to the northern borders of the Mediterranean basin. Parallel-hafted inserts, made on larger blades, became more frequent, gradually replacing serrated sickles, at least in some geographical zones, *ca*. 5300–5000 cal BCE (Fig 7c). An east-west gradient can be observed for the spread of parallel-hafted harvesting inserts, with the earliest tools documented in Greece and a gradual expansion in North Italy, Southern France and the North-East of the Iberian Peninsula. Parallel inserts were possibly dominant in Greece well before they spread west, around ca. 5900–5600, as documented in the Middle Neolithic layers of Franchthi and Sesklo [97,121]. Similar inserts, bearing parallel gloss on the used edge inserted into straight shafts, were the main type of harvesting tools in use during the PPNA [47,48], while they were substituted by parallel elements inserted into curved shafts in the PPNB. Their appearance (or re-appearance) in the Aegean area after about 2,000–2,5000 years (see also [75]) is most likely an independent innovation that attests the dynamic and changing nature of the Neolithic toolkit during the spread towards the west.

Whereas for diagonally-hafted inserts demic diffusion probably played a major role in the expansion phenomena, for parallel-hafted inserts it is more difficult to assess the mechanisms behind their adoption. The arrival of new groups, bearing new tool-types, might have played a role, at least in certain regions. In North-East Italy, for example no previous Impressed Ware occupations are known. Colonist groups would import from the Balkan area parallel-hafted blades [57], along with other cultural traits typical of the Danilo sphere (i.e. geometric incised Danilo Ware, rhyta and phalloid ceramics) [100,122,123], as well as domesticated species. New cultivars are introduced in North-East Italy, namely the ‘new glume wheat’, well known in Eastern Europe during the Early Neolithic [124,125]. In addition, the morphological traits of domesticated sheep seem to diverge between the Impressed Ware and the Cardial groups. Sheep at the Impressed Ware sites of Southern France and Central Italy are more robust and their horns are hollow, while at Cardial sites sheep are smaller and lighter, with solid horns. These latter sheep might have had a Balkan origin, arriving through North Italy [126]. It has recently also been pointed out that in the in the Ligurian-Provençal Arc a new technique for pottery manufacture appeared–the spiralled patchwork technology–, possibly related to the colonisation of the region by an unknown group, whose origins might be different from the farmer groups responsible for the colonisation of South and Central Italy, characterised by the coiling technology [127].

Other factors can be taken into account. At the time parallel-hafted tools started to be more widely adopted in the CW Mediterranean, most of the Mediterranean coasts were already settled, even if sparsely, by Neolithic pioneering groups. Technical transfers between neighbouring communities might have taken place, in addition to processes of innovation or adaptation. The gradual transition between diagonally- and parallel-hafted inserts in the Po Plain might be interpreted in the framework of a network of cultural, economic and social relationships between communities of different origins and exposed to different cultural influences [101,128]. Vice versa, the single obliquely-hafted blades recovered from La Draga might represent a case of a local, or regional, innovation, whose diffusion was initially limited to the westernmost sector of the Mediterranean arc and the interior of the Iberian Peninsula [109] between *ca*. 5420 and 5320 cal BCE (Figs 6e and 7d). Similarly, the non-use of sickles in the Cantabrian and Jura regions can be regarded as a local adaptation to particular environmental and productive conditions [110].

The adoption of parallel-hafted inserts might also be partially linked to a process of craft specialisation in lithics. As a general rule, more demanding flaking systems and flaking skills and good quality raw materials are required to produce larger and wider regular blades. Such conditions usually imply a certain degree of craft specialisation, with few knappers that are capable of producing crafts that others cannot, and final products being transferred from the producers to the consumers [129]. Nevertheless, craft specialisation is not a homogeneous process, and great differences exist between regions and periods. While specialised production characterises certain areas from the very beginning of the Neolithic (Argolis, Thessaly, South Italy, Dalmatia), other areas are dominated by domestic and largely unspecialised productions (Central Italy, Southern France, Iberian Peninsula). In addition, not only the presence of specialised craftsmen in a certain territory is relevant, but the scale of production as well. Exogenous blanks can be obtained sporadically through interchange, but not in large enough quantities to satisfy demand for harvesting inserts, which is a strategic phase in the agricultural process; this might be a plausible scenario for the pioneering seafaring groups.

Apart from aspects related to the production systems for harvesting inserts, the switch between diagonally- and parallel-hafted blades implies a change in tool maintenance strategy. Tool design, durability and maintainability are strictly interrelated factors that strongly affect productivity, especially in intense and time-consuming labour such as cereal harvesting. The cutting part of a sickle is one of the most important factors affecting the working capacity of the harvester. Working with stone inserts, cutting edges start to become dull after a dozen hours of harvesting, due to both abrasive and adhesive wear processes. Blunt edges cause a loss of cutting effectiveness, reducing harvester performance and increasing fatigue and energy demand. Different systems can be applied to maintain the sharpness of the lithic edge. As mentioned above, one is to replace used inserts with new ones. Another is through recurrent edge-retouching. Retouch removes material from—and reshapes—the cutting bevel and edge of a blade; in addition, retouch often produces a more or less pronounced denticulation on the edge that is particularly suitable for the ‘friction cutting’ of cereal crops [51,61].

Following this perspective, the observed switch in the insert-type, might also be related to a change in the cutting-edge resharpening system. Early sickles represent a relatively cheap and adaptable technology, at least in terms of insert production; nevertheless, as blank sizes are small and the exposed surface very little, edge-resharpening is generally limited. The widespread use of larger blades as inserts allowed harvesters to exploit for much longer the lithic cutting edges, as they were not obliged to discard and replace inserts so often. The greater blade width allows for a more prolonged exploitation of the edge through resharpening, making a larger surface available, while the parallel hafting allows for the exploitation of the entire length of the edge. Such intensification in blank exploitation is well testified by the more intense edge-resharpening observed on parallel-hafted inserts. This pattern has been clearly observed in the switch between Impressed Ware and Danilo inserts in Dalmatia [57]. While diagonally-hafted edge-resharpening is rare and, eventually, marginal, it is common to observe worn-out and intensively resharpened edges on parallel-hafted inserts. This pattern opposes a ‘long management’ of parallel inserts to the ‘short management’ of diagonally-hafted inserts.

Parallel-hafted inserts will become definitively predominant during Middle Neolithic cultural phases in almost the entire north-western Mediterranean Arc, in the Sepulcros de Fosas of the NE of the Iberian Peninsula, Chassey of Southern France, SMP and Lagozza cultures of North Italy, and Danilo and Hvar cultures of Dalmatia [53,57,103,105]. The need for better performing inserts that could be used for longer periods and would be easier/quicker to resharpen is an aspect that characterises later periods as well. For example, during the Late Neolithic, reaping knives, consisting of an axially hafted long blade flaked through indirect percussion or lever pressure techniques, began to be increasingly common [130,131,132]. Those blades are usually frequently resharpened through direct retouch and used for a very long time. In other instances, harvesting tools are produced from large flakes and successively shaped through bifacial or denticulate retouch. Whatever the production system, all of these inserts are usually heavily resharpened [133,134], suggesting that the need of durable and maintainable blanks remained one of the guiding criteria when choosing a blank for the production of the harvesting inserts. This raises the question of the importance of well-designed and durable harvesting tools, especially considering that the Late Neolithic and Chalcolithic is a period of supposed farming intensification in the Mediterranean, exemplified by a more systematic employment of animal traction and the diffusion of threshing boards [135,136]. Although for the Middle Neolithic, data about farming practices is still rather fragmentary at a Mediterranean scale, recent studies are indicating that early changes in farming practices were already occurring between 5800–5000 cal BC [137]. Future studies will hopefully provide more data, making it possible to relate changes in the Neolithic harvesting toolkit with shifts in the economic and social sphere.

## 5. Conclusions

Migrations, local adaptations, and the spread of technological innovations played a major role in shaping the Mediterranean Neolithic. The analysis of the harvesting toolkit reveals dynamics of continuity and change that would otherwise be difficult to detect through the analysis of other material culture (i.e. pottery or ornaments). Early seafaring farming groups shared a common harvesting technology, which they rapidly spread across the entire Mediterranean Basin, from Greece to Portugal. Although Neolithic communities knew and used different types of harvesting tools, serrated sickles were the main type of harvesting tool at the very beginning of farming expansion because of their greater adaptability and maintainability. Other tools existed, but their use was limited in the early phases of Neolithic expansion. Adaptations in the production system of the inserts and in their use pattern probably occurred in relation to lithic raw material availability and knappers’ skills, but also in relation to the scale of production and the farming techniques adopted. The successive switch, with the diffusion of parallel-hafted inserts in the north-western Mediterranean arc, is a heterogeneous phenomenon including diffusion of new groups, technical transfers, establishment of new interaction networks and new systems of lithic production, etc., with arrhythmia and differences on both geographical and chronological scales.

## Supporting information

S1 Table The table includes the studied sites, their geographical location, chronology, the repository where archaeological collections are stored, the analysed sample and the number of identified glossy blades. All dates have been calibrated with OxCal 4.3, rounded off to the nearest 50 yrs. Type- (indeterminate glossy blades are excluded) DIAG: Diagonally hafted glossy blades; PAR: Parallel hafted glossy blades; OBL: Oblique single glossy blade. Raw materials- CH: Chert; RAD: Radiolarite; OBS: Obsidian: QU: Quartz; CA: Chalcedony; IND: Indeterminate raw material. Blank type- BL: Blade; FL: Flake; Used Edges- Number of active zones used for cereal harvesting. Resh- Number and percentage of resharpened active zones.
(XLSX)

S1 File List of all ^14^C dates cited in the text. (XLSX)

S2 File List of ^14^C dates combined using the tool R_Combine, OxCal 4.3. (XLSX)

S3 File List of ^14^C dates on short-lived samples. (XLSX)

## References

[1] Brettell C, Hollifield JF. editors. *Migration Theory*: *Talking Across Disciplines*. NewYork: Rouledge; 2000.

[2] Favell A. *Immigration*, *Integration and Mobility*. Colchester: Ecpr Press; 2015.

[3] Baker BJ, Tsuda T. editors. *Migration and disruptions*: *toward a unifying theory of ancient and contemporary migrations*. Gainesville: University Press of Florida; 2015.

[4] Anthony DW. Migration in archeology: the baby and the bathwater. *American Anthropologist*. 1990; 92: 895–914.

[5] Gamble C. *Timewalkers*: *The Prehistory of Global Colonization*. Cambridge, MA: Harvard University Press; 1994.

[6] Fix A. Migration and colonisation in human microevolution Cambridge Studies in Biological and Evolutionary Anthropology, 24 Cambridge: Cambridge University Press; 1999.

[7] Beekman CS, Christensen AF. Controlling for doubt and uncertainty through multiple lines of evidence: A new look at the Mesoamerican Nahua migrations. *Journal of Archaeological Method and Theory*. 2003; 10(2): 111–164. 10.1023/A:1024519712257

[8] Franchetti MD, Spengler RN III, editors. *Mobility and ancient society in Asia and the Americas*. Switzerland: Springer International Publishing; 2015.

[9] Cowgill GL. The Debated Role of Migration in the Fall of Ancient Teotihuacan in Central Mexico In: Baker BJ, Tsuda T. editors. *Migration and disruptions*: *toward a unifying theory of ancient and contemporary migrations*. Gainesville: University Press of Florida 2015 p. 97–122.

[10] Ammerman AJ, Cavalli-Sforza LL. Measuring the rate of spread of early farming in Europe. *Man*, *New Series*. 1971; 6: 674–688.

[11] Childe VG. *The Dawn of European Civilization*. New York: Alfred A. Knopf 1958 (1925).

[12] Mazurié de Keroualin K. 2003 *Genèse et diffusion de l’agriculture en Europe*. *Agriculteurs—chasseurs—pasteurs*. Paris: Ed. Errance, coll. des Hespérides.

[13] Perlès C. Retracer des migrations préhistoriques: un cas d’étude sur la néolithisation de l’Europe In: Baussant M, Ribert E, Rivoal I, Dos Santos, I. editors. *Logiques mémorielles*, *temporalités migratoires*. Nanterre: Presses Universitaires de Paris Nanterre 2005 p. 59–93.

[14] Guilaine J. The earliest Neolithic in the West Mediterranean: a new appraisal. *Antiquity*. 1979; 53(207), 22–30. 10.1017/s0003598x00041971

[15] Zvelebil M, Dolukhanov P. The transition to farming in Eastern and Northern Europe Article. *Journal of World Prehistory*. 1991; 5(3): 233–278. 10.1007/BF00974991

[16] Bentley RA, Price TD, Lüning J, Gronenborn D, Wahl J, Fullagar PD. Prehistoric migration in Europe: strontium isotope analysis of Early Neolithic skeletons. *Current Anthropology*. 2002; 43: 799–804. 10.1086/344373

[17] Alday Ruiz A. The Transition between the Last Hunter-Gatherers and the First Farmers in Southwestern Europe: The Basque Perspective. *Journal of Anthropological Research*. 2005; 61(4): 469–494.

[18] Rowley-Conwy P. Westward Ho! The Spread of Agriculturalism from Central Europe to the Atlantic. *Current Anthropology*. 2011; 52(S4): S431–S451. 10.1086/658368

[19] Leppard TP. Mobility and migration in the Early Neolithic of the Mediterranean: questions of motivation and mechanism. *World Archaeology*. 2014; 46(4): 484–501. 10.1080/00438243.2014.931245

[20] Dennell RW. The Hunter-Gatherer/Agricultural Frontier in Prehistoric Temperate Europe In: Green WS, Perlman SM. editors. *Forager/Farmer Interactions*: *Information*, *Social Organization*, *and the Frontier*, The Archaeology of Frontiers and Boundaries. London: Academic Press 1985 p. 113–139.

[21] Bernabeu J. Indigenismo y migracionismo. Aspectos de la neolitización en la fachada oriental de la Península Ibérica. *Trabajos de Prehistoria*. 1996; 53(2): 37–54.

[22] Guilaine J. The Neolithization of Mediterranean Europe In: Fowler C, Harding J, Hofmann D, editors. *The Oxford Handbook of Neolithic Europe*. Oxford: Oxbow books 2015 p. 81–98.

[23] Gronenborn D. Mesolithic-Neolithic interactions the lithic Industry of the Earliest Bandkeramik Culture Site at Friedberg-Bruchenbrücken // Wetteraukreis (West Germany). In: Vermeersch M, Van Peer P. editors. Contributions to the Mesolithic in Europe. Papers presented at the fourth international Symposium ’The mesolithic in Europe’. Leuven: Leuven Unviersitty Press. 1990. p. 173–182.

[24] Perlès C. *Les industries lithiques taillées de Franchthi (Argolide*, *Grèce)*, vol. 2 Excavations in Franchthi Cave, Fascicule 5. Indianopolis: Indiana University Press 1990.

[25] Pedrotti A. The Neolithic Age in Trentino Alto Adige. *Preistoria Alpina*. 2001; 34: 19–25.

[26] Kaczanowska M, Kozlowski J. Lithic industry from the Aceramic levels at Knossos (Crete, Greece): An alternative approach. *Eurasian Prehistory*. 2011; 8(1–2): 67–88.

[27] Manen C, Perrin T. Réflexions sur la genèse du « Cardial franco-ibérique ». In: *De Méditerranée et d’ailleurs…Mélanges offerts à Jean Guilaine*. Toulouse: Archives d’Écologie préhistorique 2009 p. 427–443.

[28] Malone C. The Neolithic in Mediterranean Europe In: Fowler C, Harding J, Hofmann D. editors. *The Oxford Handbook of Neolithic Europe*. Oxford: Oxbow books 2015 p. 175–194.

[29] Kreuz A, Marinova E, Schäfer E, Wiethold J. A comparison of early Neolithic crop and weed assemblages from the Linearbandkeramik and the Bulgarian Neolithic cultures: differences and similarities. *Vegetation History and Archaeobotany*. 2005; 14: 237–258. 10.1007/s00334-005-0080-0

[30] Conolly J, Colledge S, Shennan S. Founder effect, drift, and adaptive change in domestic crop use in early Neolithic Europe. *Journal of Archaeological Science*, 2008; 35(10): 2797–2804. 10.1016/j.jas.2008.05.006

[31] Krauß R, Marinova E, De Brue H, Weninger B. The rapid spread of early farming from the Aegean into the Balkans via the Sub-Mediterranean-Aegean Vegetation Zone. *Quaternary International*. 2018 496: 24–41. 10.1016/j.quaint.2017.01.019

[32] Ivanova M, De Cupere B, Ethier J, Marinova E. Pioneer farming in southeast Europe during the early sixth millennium BC: Climate-related adaptations in the exploitation of plants and animals. *PlosONE*. 2018; 13(8): e0197225 https://10.1371/journal.pone.0197225. 10.1371/journal.pone.0197225 PMC5959071 29775469

[33] Vigne J-D, Briois F, Zazzo A, Willcox G, Cucchi T, Thiébault S, et al Early cultivators spread to Cyprus 10,600 y ago. *PNAS*. 2012; 109(22): 8445–8449. 22566638 10.1073/pnas.1201693109 PMC3365171

[34] Moore AMT. Agricultural origins in the Near East: A model for the 1980s. *World Archaeology*. 1982; 14(2): 224–236. 10.1080/00438243.1982.9979863

[35] Bar-Yosef O, Valla F. The Natufian Culture and the Origin of the Neolithic in the Levant. *Current Anthropology*. 1990; 31(4): 433–436. 10.1086/203867

[36] Hillman GC. The plant food economy of Abu Hureyra 1 and 2; Abu Hureyra 1: The Epipaleolithic In: Moore AMT, Hillman GC, Legge AJ, editors. *Village on the Euphrates*, *From foraging to farming at Abu Hureyra*. Oxford: Oxford University Press 2000 p. 327–398.

[37] Willcox G, Stordeur D. Large-scale cereal processing before domestication during the tenth millennium cal BC in northern Syria. *Antiquity*. 2012; 86(331): 99–114. 10.1017/S0003598X00062487

[38] Ibáñez JJ, Anderson PC, Gonzalez-Urquijo JE, Gibaja JF. 2016. Cereal cultivation and domestication as shown by microtexture analysis of sickle gloss through confocal microscopy. *Journal of Archaeological Science*. 2016; 73: 62–81. 10.1016/j.jas.2016.07.011

[39] Astruc L. L’outillage lithique taillé de Khirokitia *Analyse fonctionnelle et spatiale*. Monographie du CRA, 25 Paris: CNRS Éditions; 2002.

[40] Borrell F. Further remarks about lithic production at Akarçay Tepe (middle Euphrates valley) during the Late PPNB In: Affanni G, Baccarin C, Cordera L, Di Michele A, Gavagnin K. editors. *Broadening Horizons 4*. BAR International Series, Vol. 2698 Oxford: Archaeopress 2015 p. 265–278.

[41] Philibert S. Approche fonctionnelle des outillages en pierre taillée In: Guilaine J, Briois F, Vigne J-D. editors. *Shillourokambos*, *un établissement néolithique précéramique à Chypre*. *Fouilles du secteur 1*. Paris: Editions Errance 2011 p. 689–705.

[42] Gopher A, Barkai R, Asaf A. Trends in Sickle Blades Production in the Neolithic of the Hula valley, Israel In: Caneva I, Lemorini C, Zampetti D, Biagi P. editors. *Beyond Tools*: *Redefining the PPN Lithic Assemblages of the Levant*. Studies in Early Near Eastern Production, Subsistance and Environment 9. Berlin: Ex oriente 2001 p. 411–425.

[43] Bar-Yosef O. Direct and indirect evidence for hafting in the Epi-Palaeolithic and Neolithic of the Southern Levant. *Travaux de la Maison de l’Orient*. 1987; 15(1): 155–164.

[44] Edwards P. A 14000 year-old hunter-gatherer’s toolkit. *Antiquity*. 2007; 81(314): 865–876. 10.1017/S0003598X0009596X

[45] Borrell F, Molist M. Projectile Points, Sickle Blades and Glossed Points. Tools and Hafting Systems at Tell Halula (Syria) during the 8th millennium cal. BC. *Paléorient*. 2007; 33(2): 59–77. 10.3406/paleo.2007.5221

[46] Ibáñez JJ, González-Urquijo JE, Rodríguez-Rodríguez A. The evolution of technology during the PPN in the Middle Euphrates. A view from use wear analysis of lithic tools In: L, Binder D, Briois F. editors. *Systèmes techniques et communautés du Néolithique précéramique au Proche-Orient*. Antibes: Éditions APDCA 2007 p. 153–165.

[47] Pichon F. Exploitation of the cereals during the Pre-pottery Neolithic of Dja’de-el-Mughara: Preliminary results of the functional study of the glossy blades. *Quaternary International*. 2017; 427: 138–151. 10.1016/j.quaint.2016.01.064

[48] Astruc L, Briois F. Harvesting tools during the Pre-Pottery Neolithic in Cyprus In: Astruc L, Briois F, editors. *Near Eastern Lithic Technologies on the Move*. *Interactions and Contexts in Neolithic Traditions*. Studies in Mediterranean Archaeology, vol CL Nicosia: Astrom Editions 2019, p. 53–62.

[49] Bezić A. Distribution of flint in Turkey from 10,000 to 6,000 cal BC. Case study–Çatalhöyük In: Delage D. editor. *Chert Availability and Prehistoric Exploitation in the Near East*. British Archaeological Reports International Series, 1615 Oxford: Archaeopress 2007 p. 68–86.

[50] Quintero LA. Flint Mining in the Pre-Pottery Neolithic: Preliminary Report on the Exploitation of Flint at Neolithic ‘Ain Ghazal in Highland Jordan In: Kozlowski SK, Gebel HGK. editors. *Neolithic Chipped Lithic Industries of the Fertile Crescent and their Adjacent Regions*, Studies in Early Near Eastern Production, Subsistence, and Environment, 3 Berlin: Ex Oriente 1996 p. 233–260.

[51] Astruc L. Tkaya MB, Torchy L. De l’efficacité des faucilles néolithiques au Proche-Orient: approche expérimentale. *Bulletin de la Société préhistorique française*. 2012; 109(4): 671–687.

[52] Ibáñez JJ, Clemente Conte I, Gassin B, Gibaja JF, Gonzáles Urquijo, JE, Márquez B, et al Harvesting technology during the Neolithic in south-west Europe In: Longo L, Skakun N. editors. *Prehistoric technology 40 years later*: *functional studies and the Russian legacy*, British Archaeological Reports International Series, 1783 Oxford: Archaeopress 2008 p. 183–95.

[53] Gassin B, Astruc L, Bouby L, Buxo R, Clemente I, Gibaja JF, et al Variabilité des techniques de récolte et traitement des céréales dans l’Occident méditerranéen au Néolithique ancien et moyen: facteurs environnementaux, économiques et sociaux In: Beeching A, Thirault É, Vital J. editors.*7e Rencontres méridionales de Préhistoire récente*. DARA 34 Lyon: ALPARA 2010; p. 19–37.

[54] Gibaja JF, Ibáñez JJ, González Urquijo J. Neolithic Sickles in the Iberian Peninsula In: van Gijn A, Whittaker P, Anderson P. editors. *Exploring and Explaining Diversity in Agricultural Technology*, EARTH 2 Oxford: Oxbow Books 2014 p. 112–118.

[55] Ibáñez JJ, Gibaja JF, Gassin B, Mazzucco N. Paths and rhythms in spread of agriculture in the Western Mediterranean: the contribution of the analysis of harvesting technology In: Salazar-García DC, García-Puchol O. editors. *Times of Neolithic Transition along the Western Mediterranean*. Springer Series Fundamental Issues in Archaeology. New York: Springer. 2017 p. 339–371.

[56] Mazzucco N, Capuzzo G, Petrinelli-Pannocchia C, Ibáñez JJ, Gibaja JF. Harvesting tools and the spread of the Neolithic into the Central-Western Mediterranean area. *Quaternary International*. 2018; 470(Part B): 511–528. 10.1016/j.quaint.2017.04.018

[57] Mazzucco N, Guilbeau D, Kačar S, Podrug E, Forenbaher S, Radić D, et al The time is ripe for a change. The evolution of harvesting technologies in Central Dalmatia during the Neolithic period (6th millennium cal BC). *Journal of Anthropological Archaeology*. 2018; 51: 88–103. 10.1016/j.jaa.2018.06.003

[58] Boserup E. The Conditions of Agricultural Progress. Chicago: Aldine Publishing Company, 1965.

[59] Sigaut F. Identification des techniques de récolte des graines alimentaires. *Journal d’agriculture traditionnelle et de botanique appliquée*. 1978; 25(3): 145–161.

[60] Comet G. *Le Paysan et son outil*. *Essai d’histoire technique des céréales (France*, *VIIIe-XVe siècle)*. Vol. 165 No. 1. Rome: Ecole française de Rome; 1992.

[61] Anderson PC, Sigaut F. Introduction: reasons for variability in harvesting techniques and tools In: van Gijn A, Whittaker J, Anderson P, editors. *Exploring and Explaining Diversity in Agricultural Technology*, Earth, vol. 2 Oxford: Oxbow Books; 2014 p. 85–93.

[62] Halstead P. Two oxen ahead: pre-mechanized farming in the Mediterranean. Chichester: John Wiley & Sons; 2014.

[63] Inizan ML. Séries anciennes et économie du débitage In: *Préhistoire et technologie lithique*, Publications de l’URA 28, Cahier 1 du CRA. Paris: Éditions du CNRS; 1980 p. 28–30.

[64] Soressi M, Geneste J-M. The history and efficacy of the Chaîne Opératoire approach to lithic analysis: studying techniques to reveal past societies in an evolutionary perspective In: Tostevin GB. editor. *Reduction Sequence*, *Chaîne Opératoire and Other Methods*: *The Epistemologies of Different Approaches to Lithic Analysis*. *PaleoAnthropology*. 2001; Special Issue: 334–350.

[65] Ibáñez JJ, González-Urquijo JE, Gibaja JF. Discriminating wild vs domestic cereal harvesting micropolish through laser confocal microscopy. *Journal of Archaeological Science*, 2014; 48, 96–103.

[66] Bronk Ramsey C. Bayesian analysis of radiocarbon dates. Radiocarbon. 2009; 51(1): 337–60.

[67] Feeser I, Dörfler W, Kneisel J, Hinz M, Dreibrodt S. Human impact and population dynamics in the Neolithic and Bronze Age: Multi-proxy evidence from north-western Central Europe. *The Holocene*. 2019; 0959683619857223.

[68] Loftus E, Mitchell PJ, Ramsey CB. An archaeological radiocarbon database for southern Africa. *Antiquity*. 2019; 93(370), 870–885.

[69] McLaughlin TR. On applications of space–Time modelling with open-source 14 C Age calibration. *Journal of Archaeological Method and Theory*. 2019; 26(2), 479–501.

[70] Bronk Ramsey C. Methods for summarizing radiocarbon datasets. *Radiocarbon*. 2017; 59(6): 1809–1833.

[71] Perlès C. *The early Neolithic in Greece*: *the first farming communities in Europe*. Cambridge: Cambridge University Press; 2001.

[72] Douka K, Efstratiou N, Hald MM, Henriksen PS, Karetsou A. Dating Knossos and the arrival of the earliest Neolithic in the southern Aegean. *Antiquity*. 2017; 91(356): 304–321. 10.15184/aqy.2017.29

[73] Efstratiou N, Karetsou A, Banou ES, Margomenou D. The Neolithic settlement of Knossos: new light on an old picture In: *British School at Athens Studies*, Vol 12 Knossos: Palace, City, State Athens: British School at Athens 2004 p. 39–49.

[74] Conolly J. The knapped stone technology of the first occupants at Knossos In: Isaakidou V, Tomkins P. editor. *Escaping the labyrinth*: *the Cretan Neolithic in context*. Sheffield Studies in Aegean Archaeology, Vol. 8 Oxford: Oxbow Books 2008 p. 73–89.

[75] Guilbeau D, Kayacan N, Altınbilek-Algül Ç, Erdoğu B, Çevik Ö. A comparative study of the Initial Neolithic chipped-stone assemblages of Ulucak and Uğurlu. *Anatolian Studies*. 2019; 69: 1–20.

[76] Phlourentzos P, McCartney C, Croft P, Reese DS, Ergōn CHS, Archaiotētōn CT. *The Neolithic Settlement of Paralimni*. Cyprus: Dept. of Antiquities; 2008.

[77] Perlès C. Grece et Balkans: deux voies de penetration distinctes du Neolithique en Europe? In: Demoule J-P, editor. *La revolution neolithique dans le monde*. Paris: CNRS Editions 2010 p. 263–281.

[78] Burmeister S. Archaeology and migration: approaches to an archaeological proof of migration. *Current Anthropology*. 2002; 41(4): 539–567. 10.1086/317383

[79] Costa L-J. Récents acquis sur la circulation préhistorique de l’obsidienne en Corse. *Bulletin de la Société préhistorique française*. 2006; 103(1): 71–85.

[80] Cappai R. L’industria litica dalle domus de janas III e IV: un esempio di gestione integrata delle risorse In: Melis MG. Editor. *Usini*. *Ricostruire il passato*. *Una ricerca internazionale a S’Elighe Entosu*. Sassari: Carlo Delfino Ed 2010 p. 219–236.

[81] Rodríguez Rodríguez AC, Hernández Gómez M. ‘Lágrimas negras’. L’exploitation de l’obsidienne aux Îles Canaries: de la simplicité des systèmes de taille à la spécialisation artisanale In: Astruc L, Bon F, Léa V, Milcent P-Y, Philibert S. editors. *Normes techniques et pratiques sociales*. *De la simplicité des outillages pré- et protohistoriques*. XXVIe rencontres internationales d’archéologie et d’histoire d’Antibes. Antibes: Éditions APDCA 2006 p. 393–403.

[82] Bogaard A, Halstead P. Subsistence Practices and Social Routine in Neolithic Southern Europe In: The *Oxford Handbook of Neolithic Europe*. Fowler C. Harding J. and Hofmann D. editors. Oxford: Oxford University Press 2015 p. 362–385.

[83] Forenbaher S, Timothy K, Preston TM. Dating the East Adriatic Neolithic." European Journal of Archaeology. 2013; 16(4): 589–609.

[84] Gabriele M, Convertini F, Vérati C, Gratuze B, Jacomet, S, Boschian G, et al Long-distance mobility in the North-Western Mediterranean during the Neolithic transition using high resolution pottery sourcing. *Journal of Archaeological Science*: Reports. 2019; 28: 102050.

[85] Guilaine J, Manen C. From Mesolithic to early Neolithic in the western Mediterranean In: Whittle A, Cummings V. editors. *Going over*: *the Mesolithic-Neolithic transition in North-West Europe*. Oxford: Oxford University Press 2007 p. 21–51.

[86] Binder D, Lanos P, Angeli L, Gomart L, Guilaine J, Manen C, et al Modelling the earliest north-western dispersal of Mediterranean Impressed Wares: new dates and Bayesian chronological model. *Documenta praehistorica*. 2017; 44: 54–77. doi: 10.4312\dp.44.4

[87] Buxó R, Rovira N, Sauch C. Les restes vegetals de llavors i fruits In: Bosch A, Chinchilla J, Tarrús J. Editors. *El poblat lacustre neolític de la Draga*. *Excavacions de 1990 a 1998*. Monografies del CASC, 2 Girona: Museu d’Arqueologia de Catalunya 2000 p. 129–140.

[88] Rottoli M. Crop Diversity Between Central Europe and the Mediterranean: Aspects of Northern Italian Agriculture In: Chevalier A, Marinova E, Peña-Chocarro L. editors. *Plants and People*: *Choices and Diversity Through Time*. EARTH, vol 1 Oxford: Oxbow Books 2013 p. 90–104.

[89] Reed K, Colledge S. Plant Economies in the Neolithic Eastern Adriatic: Archaeobotanical Results from Danilo and Pokrovnik. *Vjesnik za Arheologiju i Povijest Dalmatinsku*. 2016; 1(9): 9–23.

[90] Pérez-Jordà G, Peña-Chocarro L, Morales M. Agricultura neolítica en Andalucía: semillas y frutos. *Menga*: *Revista de prehistoria de Andalucía*. 2011; 2: 59–72.

[91] Borrell M, Borrell F, Bosch J, Clop X, Molist M. editors. Xarxes al Neolític. Circulació i intercanvi de matèries, productes i idees a la Mediterrània occidental (VII-III mil·lenni aC) Gavà / Bellaterra, 2–4 / 2 / 2011. Rubricatum: revista del Museu de Gavà, 5; 2012.

[92] Zapata L, Peña-Chocarro L, Pérez-Jordá G, Stika HP. Early Neolithic agriculture in the Iberian Peninsula. *Journal of world Prehistory*. 2004; 18(4): 283–325. 10.1007/s10963-004-5621-4

[93] Gurova M. “Cereal polish”: Diagnosis, Challenge or Confusion. In: Marreiros J, Bicho N, Gibaja JF. editors. International Conference on Use-Wear Analysis, Use-Wear 2012. Newcastle upon Tyne: Cambridge Scholars Publishing. 2014. p. 90–102.

[94] Gurova M. Prehistoric sickles in the collection of the National Museum of Archaeology in Sofia In: Bacvarov K, Gleser E, editors. Southeast Europe and Anatolia in Prehistory: Essays in Honor of Vassil Nikolov on his 65th Anniversary. Bonn: Verlag Dr. Rudolf Habelt GmbH 2016 p. 159–165.

[95] Bleed P. The optimal design of hunting weapons: maintainability or reliability. *American antiquity*, 1986; 51(4): 737–747. 10.2307/280862

[96] Perlès C, Vaughan P. Pièces lustrées, travail des plantes et moissons à Franchthi (Grèce) (Xème-IVème mill. B.C.) In: Cauvin M-P. editor. *Traces d’utilisation sur les outils néolithiques du Proche-Orient*. Travaux de la Maison de l’Orient méditerranéen, 5 Lyon: MOM Éditions 1983 p. 209–224.

[97] Perlès C. Les industries lithiques taillées de Franchthi (Argolide, Grèce). Tome III, Du Néolithique ancien au Néolithique final Excavations at Franchthi cave, Greece, 13 Bloomington and Indianapolis: Indiana University Press; 2004.

[98] Maniatis Y, Kotsakis K, Halstead P. Paliambela Kolindrou. Nees chronologēseis tēs archaēoteris Neolithikēs. In: Archaiologiko Ergo stē Makedonia kai Thrakē 25. 2015. p. 149–156.

[99] Korona M. Kremeni artefakti In: Marijanović B. editor. Crno Vrilo 2. Zadar: Sveučilište u Zadru, 2009 p. 145–218.

[100] Mazzucco N, Gibaja JF, Pessina A, Ibáñez JJ. Reconstructing harvesting technologies through the analysis of sickle blades: a case-study from Early-Middle Neolithic sites in northeastern Italy. *Lithic Technology*. 2016; 41(1): 75–92. 10.1080/01977261.2016.1149654

[101] Starnini E, Biagi P, Mazzucco N. The beginning of the Neolithic in the Po Plain (northern Italy): Problems and perspectives. *Quaternary International*. 2018; 470: 301–317. 10.1016/j.quaint.2017.05.059

[102] Maggi R, Starnini E, Voytek B. Arene Candide: a functional and environmental assessment of the Holocene sequence excavated by L. Bernabò Brea (1940–50) Memorie dell’Istituto Italiano di Paleontologia Umana, Vol. 5 Roma: Istituto Italiano di Paleontologia Umana; 1997.

[103] Biagi P, Nisbet R. The Earliest Farming Communities in Northern Italy In: Guilaine J. editor. *Premières communautés paysannes en Méditerranée occidentale*. Paris: CNRS Éditions; 1987 p. 449–453

[104] De Stefanis C. Systèmes techniques des derniers chasseurs-cueilleurs et des premiers agro-pasteurs du domaine liguro-provençal (7000–5500 av. JC): approche fonctionnelle. PhD dissertation, Université Côte d’Azur, Nice; 2018.

[105] Gibaja JF. Las hoces neolíticas del noreste de la península ibérica. *Préhistoire Anthropologie méditerranéennes*. 2002; 10–11: 83–96. journals.openedition.org/pm/254.

[106] Domingo R. Beyond Chaves: Functional Analysis of Neolithic Blades from the Ebro Valley. In: Marreiros J, Bicho N, Gibaja JF. editors. International Conference on Use-Wear Analysis, Use-Wear 2012. Newcastle upon Tyne: Cambridge Scholars Publishing. 2014. p. 672–681.

[107] Palomo A. Gibaja JF, Piqué R, Bosch A, Chinchilla J, Tarrús J. Harvesting cereals and other plants in Neolithic Iberia: The assemblage from the lake settlement at La Draga. *Antiquity*. 2011; 85: 759–771. 10.1017/S0003598X00068290

[108] Flors E. Gibaja JF, Ibáñez JJ, Salazar D. Neolithic Communities on the Mediterranean Coast: a new antler sickle handle from Costamar (Cabanes, Castellón, Spain). Antiquity. 2012; 86/332.

[109] Gibaja JF. Ibáñez JJ, Nielsen E. Kienholz A. van Willigen S, Linton J. The Neolithic reaping knives from Egolzwil 3: A Mediterranean technical tradition in the late 5th millennium Swiss Neolithic. *Quaternary International*. 2017; 427: 211–224. 10.1016/j.quaint.2015.12.075

[110] Ibáñez JJ, González-Urquijo JE, Peña-Chocarro L, Zapata L, Beugnier V. Harvesting without sickles. Neolithic examples from humid mountain areas In: Beyries S, Petrequin P. editors. *Ethno-Archaeology and its Transfers*. British Archaeological Reports International Series, 983 Oxford: Archaeopress 2001 p. 23–36.

[111] Ortiz F, Sigaut F. La moisson de l’épeautre avec les “mesorias” dans deux villages asturiens. *Bulletin de la Societé d’Ethnozoologie et d’Ethnobotanique*. 1980; 8: 2–4.

[112] Steele J, Glatz C, Kandler A. Ceramic diversity, random copying, and tests for selectivity in ceramic production. *Journal of Archaeological Science*. 2010; 37(6): 1348–1358. 10.1016/j.jas.2009.12.039

[113] Shennan SJ, Crema ER, Kerig T. Isolation-by-distance, homophily, and “core” vs. “package” cultural evolution models in Neolithic Europe. *Evolution and Human Behavior*. 2015; 36(2): 103–109. 10.1016/j.evolhumbehav.2014.09.006

[114] Bernabeu J, Lozano S, Pardo-Gordó S. Iberian Neolithic Networks: The Rise and Fall of the Cardial World. *Frontiers in Digital Humanities*. 2017; 4 10.3389/fdigh.2017.00007

[115] Ibáñez JJ, González-Urquijo J, Teira-Mayolini LC, Lazuén T. The emergence of the Neolithic in the Near East: A protracted and multi-regional model *Quaternary International*. 2018; 470(Part B): 226–252. 10.1016/j.quaint.2017.09.040

[116] Çilingiroğlu Ç. The concept of “Neolithic package”: considering its meaning and applicability. *Documenta Praehistorica*. 2005; 32: 1–13. 10.4312/dp.32.1

[117] Perlès C. Tempi of change: When soloists don’t play together. Arrhythmia in ‘continuous’ change. *Journal of Archaeological Method and Theory*. 2013; 20(2): 281–299.

[118] Rigaud S, Manen C, García-Martínez de Lagrán I. Symbols in motion: Flexible cultural boundaries and the fast spread of the Neolithic in the western Mediterranean. *PLoS ONE*. 2018; 13(5): e0196488 10.1371/journal.pone.0196488 29715284 PMC5929525

[119] Manen C. Structure et identité des styles céramiques du Néolithique ancien entre Rhône et Èbre. *Gallia Préhistoire*. 2002; 44: 121–165.

[120] Guilaine J. La diffusion de l’agriculture en Europe: une hypothèse arythmique. Zephyrus: Revista de prehistoria y arqueología 2000; 53: 267–272.

[121] Moundrea-Agrafioti A. Pièces lustrées du Néolithique thessalien: essai de classement In: *Traces d’utilisation sur les outils néolithiques du Proche Orient*. Travaux de la Maison de l’Orient, 5 Lyon: Maison de l’Orient et de la Méditerranée Jean Pouilloux, 1983 p. 199–207.

[122] Biagi P. The Rhyton of the Balkan Peninsula: Chronology, Origin, Dispersion and Function of a Neolithic "Cult" Vessel. *Journal of Prehistoric Religion*. 2003; 16/17: 16–26.

[123] Pessina A. Nuovi dati sugli aspetti culturali del primo Neolitico in Friuli e sui rapporti con l’adriatico orientale In: Pessina A, Paola V. editors. *Preistoria dell’Italia settentrionale*. *Studi in ricordo di Bernardo Bagolini*. *Atti del Convegno*. Udine: Edizioni del Museo Friulano di Storia Naturale; 2006 p. 279–302.

[124] Jones GEM, Valamoti S, Charles M. Early crop diversity: A "new" glume wheat from northern Greece. *Vegetation History and Archaeobotany*. 2000; 9(3): 133–146. 10.1007/BF01299798

[125] Rottoli M, Castiglioni E. Prehistory of plant growing and collecting in northern Italy, based on seed remains from the Early Neolithic to the Chalcolithic (c. 5600–2100 cal BC). *Vegetation History and Archaeobotany*. 2009; 18: 91–103. 10.1007/s00334-007-0139-1

[126] Vigne J-D. Exploitation des animaux et néolithisation en Méditerranée nord-occidentale In: Guilaine J. Manen C, Vigne J-D. editors. *Pont de Roque-Haute (Portiragnes*, *Hérault)*. *Nouveaux regards sur la néolithisation de la France méditerranéenne*.. Toulouse: Archives d’Ecologie Préhistorique; 2007 p. 221–31.

[127] Gomart L, Weiner A, Gabriele M, Durrenmath G, Sorin S, Angeli L, et al Spiralled patchwork in pottery manufacture and the introduction of farming to Southern Europe. *Antiquity*. 2017; 91(360): 1501–1514.

[128] Perrin T. New Perspectives on the Mesolithic/Neolithic Transition in Northern Italy In: McCartan S, Schulting R, Warren G, Woodman P. editors. *Mesolithic Horizons*. Belfast: Oxbow Books; 2009 p. 514–520.

[129] Perlès C, Vitelli KD. Craft specialisation in the Neolithic of Greece In: Halstead P. editor. *Neolithic society in Greece*. Sheffield Studies in Aegean Archaeology. Sheffiled: Sheffiled Academic Press Ltd; 1999 p. 96–107.

[130] Plisson H, Mallet N, Bocquet A, Ramseyer D. 2002. Utilisation et rôle des outils en silex du Grand-Pressigny dans les villages de Charavines et de Portalban (Néolithique final). *Bulletin de la Société préhistorique française*. 2002; 99(99): 793–811.

[131] Gibaja JF, Terradas X, Palomo A, Clop X. Las grandes láminas de sílex documentadas en contextos funerarios del Neolítico final-Bronce inicial en el nordeste peninsular In: Gibaja JF, Terradas X, Palomo A, Clops X. editors. Les grans fulles de sílex. Europa al final de la Prehistòria. Barcelona: Museu d’Arqueologia de Catalunya; 2009 p. 63–68.

[132] Anzidei PA, Carboni G, Carboni L, Catalano P, Celant A, Cereghino R, et al Il Gaudo a Sud del Tevere: abitati e necropoli dall’area romana In: *Atti della XLIII Riunione Scientifica IIPP*, *L’età del Rame in Italia*. Firenze: I.I.P.P; 2011 p. 309–321.

[133] Sestier C. Méthode d’étude de la retouche des outils sur lames de silex pressigniennes. Critères techno-fonctionnels quantitatifs et qualitatifs. *ArcheoSciences*. *Revue d’archéométrie*. 2010; 34: 9–24.

[134] Linton J. Analyse technique et fonctionnelle de l’outillage en silex du Grand-Pressigny au Néolithique récent et final de la Touraine au plateau Suisse: résumé de thèse. *Bulletin des amis du Musée de Préhistoire du Grand-Pressigny*. 2012; 63: 7–10.

[135] Anderson PC, Chabot HTJ, van Gijn AL. The funcional riddle of ’glossy’ Canaanean blades and the Near Eastern threshing sledge. *Journal of Mediterranean Archaeology*. 2004; 17: 44.

[136] Gibaja JF, Crespo M, Delibes de Castro G, Fernández Manzano J, Fraile C, Herrán Martínez JI, et al EL uso de trillos durante la Edad del Cobre en la Meseta española. Análisis traceológico de una colección de denticulados de sílex procedentes del ‘recinto de fosos’ de El Casetón de la Era (Villalba de los Alcores, Valladolid). *Trabajos de prehistoria*. 2012; 69(1): 133–148.

[137] Vaiglova P, Bogaard A, Collins M, Cavanagh W, Mee C, Renard J, et al An integrated stable isotope study of plants and animals from Kouphovouno, southern Greece: a new look at Neolithic farming. *Journal of Archaeological Science*. 2014; 42: 201–215. 10.1016/j.jas.2013.10.023

