# Correction: Migration, adaptation, innovation: The spread of Neolithic harvesting technologies in the Mediterranean

**DOI:** 10.1371/journal.pone.0235874

**Published:** 2020-07-02

**Authors:** Niccolò Mazzucco, Juan José Ibáñez, Giacomo Capuzzo, Bernard Gassin, Mario Mineo, Juan Francisco Gibaja

In the 3.1 Going westward: The Aegean Sea and mainland Greece subsection of the 3. Results section, there is an error in the first sentence of the second paragraph. The authors did not analyze materials from the 1997 excavation. The authors received permission to study the 1957–60 and 1969–70 assemblage via permit KNO-12 issued by the British School of Athens. The correct sentence is: The flaked stone assemblage recovered from the 1957–60 and 1969–70 excavation campaigns by John Evans in the Palace of Knossos has been fully analysed in the frame of this research (S1 Table), offering the earliest evidence of the use of harvesting tools in the Aegean.
